# Circulating bilirubin levels and risk of colorectal cancer: serological and Mendelian randomization analyses

**DOI:** 10.1186/s12916-020-01703-w

**Published:** 2020-09-03

**Authors:** Nazlisadat Seyed Khoei, Mazda Jenab, Neil Murphy, Barbara L. Banbury, Robert Carreras-Torres, Vivian Viallon, Tilman Kühn, Bas Bueno-de-Mesquita, Krasimira Aleksandrova, Amanda J. Cross, Elisabete Weiderpass, Magdalena Stepien, Andrew Bulmer, Anne Tjønneland, Marie-Christine Boutron-Ruault, Gianluca Severi, Franck Carbonnel, Verena Katzke, Heiner Boeing, Manuela M. Bergmann, Antonia Trichopoulou, Anna Karakatsani, Georgia Martimianaki, Domenico Palli, Giovanna Tagliabue, Salvatore Panico, Rosario Tumino, Carlotta Sacerdote, Guri Skeie, Susana Merino, Catalina Bonet, Miguel Rodríguez-Barranco, Leire Gil, Maria-Dolores Chirlaque, Eva Ardanaz, Robin Myte, Johan Hultdin, Aurora Perez-Cornago, Dagfinn Aune, Konstantinos K. Tsilidis, Demetrius Albanes, John A. Baron, Sonja I. Berndt, Stéphane Bézieau, Hermann Brenner, Peter T. Campbell, Graham Casey, Andrew T. Chan, Jenny Chang-Claude, Stephen J. Chanock, Michelle Cotterchio, Steven Gallinger, Stephen B. Gruber, Robert W. Haile, Jochen Hampe, Michael Hoffmeister, John L. Hopper, Li Hsu, Jeroen R. Huyghe, Mark A. Jenkins, Amit D. Joshi, Ellen Kampman, Susanna C. Larsson, Loic Le Marchand, Christopher I. Li, Li Li, Annika Lindblom, Noralane M. Lindor, Vicente Martín, Victor Moreno, Polly A. Newcomb, Kenneth Offit, Shuji Ogino, Patrick S. Parfrey, Paul D. P. Pharoah, Gad Rennert, Lori C. Sakoda, Clemens Schafmayer, Stephanie L. Schmit, Robert E. Schoen, Martha L. Slattery, Stephen N. Thibodeau, Cornelia M. Ulrich, Franzel J. B. van Duijnhoven, Korbinian Weigl, Stephanie J. Weinstein, Emily White, Alicja Wolk, Michael O. Woods, Anna H. Wu, Xuehong Zhang, Pietro Ferrari, Gabriele Anton, Annette Peters, Ulrike Peters, Marc J. Gunter, Karl-Heinz Wagner, Heinz Freisling

**Affiliations:** 1 Department of Nutritional Sciences, Faculty of Life Sciences, University of Vienna, Vienna, Austria; 2 Nutritional Epidemiology Group, Section of Nutrition and Metabolism, International Agency for Research on Cancer (IARC-WHO), Lyon, France; 3 Public Health Sciences Division, Fred Hutchinson Cancer Research Center, Seattle, WA USA; 4 Colorectal Cancer Group, ONCOBELL Program, Bellvitge Biomedical Research Institute (IDIBELL). L’Hospitalet de Llobregat, Barcelona, Spain; 5 Nutritional Methodology and Biostatistics Group, Section of Nutrition and Metabolism, International Agency for Research on Cancer (IARC-WHO), 150 cours Albert Thomas, 69372 Lyon CEDEX 08, France; 6 Division of Cancer Epidemiology, German Cancer Research Center (DKFZ), Heidelberg, Germany; 7 Department for Determinants of Chronic Diseases (DCD), National Institute for Public Health and the Environment (RIVM), Bilthoven, The Netherlands; 8 Department of Gastroenterology and Hepatology, University Medical Center, Utrecht, The Netherlands; 9 Department of Epidemiology and Biostatistics, School of Public Health, Imperial College London, London, UK; 10 Department of Social and Preventive Medicine, Faculty of Medicine, University of Malaya, Kuala Lumpur, Malaysia; 11 Group Nutrition, Immunity and Metabolism, Department of Nutrition and Gerontology, German Institute of Human Nutrition Potsdam-Rehbruecke, Nuthetal, Germany; 12 International Agency for Research on Cancer (IARC-WHO), Lyon, France; 13 School of Medicine, Griffith University, Brisbane, QLD Australia; 14 Alliance for Vascular Access Teaching and Research (AVATAR), Menzies Health Institute Queensland, Griffith University, Brisbane, QLD Australia; 15 Danish Cancer Society Research Center, Copenhagen, Denmark; 16 Department of Public Health, University of Copenhagen, Copenhagen, Denmark; 17 CESP (Centre de Recherche en Epidémiologie et Santé des Populations), Fac. de médecine - Univ. Paris-Sud, Fac. de médecine - UVSQ, INSERM, Université Paris-Saclay, Villejuif, France; 18 Institut Gustave Roussy, Villejuif, France; 19 Department of Gastroenterology, Bicêtre University Hospital, Public Assistance Hospitals of Paris, Le Kremlin Bicêtre, France; 20 Department of Epidemiology, German Institute of Human Nutrition Postdam-Rehbrücke, Nuthetal, Germany; 21 Hellenic Health Foundation, Athens, Greece; 22 2nd Pulmonary Medicine Department, School of Medicine, National and Kapodistrian University of Athens, “ATTIKON” University Hospital, Haidari, Greece; 23 Cancer Risk Factors and Life-Style Epidemiology Unit, Institute for Cancer Research, Prevention and Clinical Network – ISPRO, Florence, Italy; 24 Lombardy Cancer Registry Unit, Fondazione IRCCS Istituto Nazionale dei Tumori, Milan, Italy; 25 Dipartimento di Medicina Clinica e Chirurgia, Federico II University, Naples, Italy; 26 Cancer Registry and Histopathology Department, “M.P. Arezzo” Hospital, ASP Ragusa, Ragusa, Italy; 27 Unit of Cancer Epidemiology, Città della Salute e della Scienza University-Hospital and Center for Cancer Prevention (CPO), Turin, Italy; 28 Department of Community Medicine, Faculty of Health Sciences, University of Tromsø (UiT), The Arctic University of Norway, Tromsø, Norway; 29 Nutritional Epidemiology Group, School of Food and Nutrition, University of Leeds, Leeds, UK; 30 Public Health Directorate, Asturias, Spain; 31 Cancer Epidemiology Research Program, Unit of Nutrition and Cancer, Catalan Institute of Oncology (ICO-IDIBELL), Barcelona, Spain; 32 Escuela Andaluza de Salud Pública. Instituto de Investigación Biosanitaria, ibs. GRANADA, Universidad de Granada, Granada, Spain; 33 CIBER in Epidemiology and Public Health (CIBERESP), Madrid, Spain; 34 Public Health Division of Gipuzkoa-BIODONOSTIA, Basque Regional Health Department, San Sebastian, Spain; 35 Department of Epidemiology, Regional Health Council, IMIB-Arrixaca, Murcia University, Murcia, Spain; 36 Navarra Public Health Institute, Pamplona, Spain; 37 IdiSNA, Navarra Institute for Health Research, Pamplona, Spain; 38 Department of Radiation Sciences, Oncology Unit, Umeå University, Umeå, Sweden; 39 Department of Medical Biosciences, Clinical Chemistry, Umeå University, Umeå, Sweden; 40 Cancer Epidemiology Unit, Nuffield Department of Population Health, University of Oxford, Oxford, UK; 41 Department of Nutrition, Bjørknes University College, Oslo, Norway; 42 Department of Endocrinology, Morbid Obesity and Preventive Medicine, Oslo University Hospital, Oslo, Norway; 43 Department of Hygiene and Epidemiology, University of Ioannina School of Medicine, Ioannina, Greece; 44 Division of Cancer Epidemiology and Genetics, National Cancer Institute, National Institutes of Health, Bethesda, MD USA; 45 Department of Medicine, University of North Carolina School of Medicine, Chapel Hill, NC USA; 46 Service de Génétique Médicale, Centre Hospitalier Universitaire (CHU) Nantes, Nantes, France; 47 Division of Clinical Epidemiology and Aging Research, German Cancer Research Center (DKFZ), Heidelberg, Germany; 48 Division of Preventive Oncology, German Cancer Research Center (DKFZ) and National Center for Tumor Diseases (NCT), Heidelberg, Germany; 49 German Cancer Consortium (DKTK), German Cancer Research Center (DKFZ), Heidelberg, Germany; 50 Behavioral and Epidemiology Research Group, American Cancer Society, Atlanta, GA USA; 51 Center for Public Health Genomics, University of Virginia, Charlottesville, VA USA; 52 Division of Gastroenterology, Massachusetts General Hospital and Harvard Medical School, Boston, MA USA; 53 Channing Division of Network Medicine, Brigham and Women’s Hospital and Harvard Medical School, Boston, MA USA; 54 Clinical and Translational Epidemiology Unit, Massachusetts General Hospital and Harvard Medical School, Boston, MA USA; 55 Broad Institute of MIT and Harvard, Cambridge, MA USA; 56 Department of Epidemiology, Harvard T.H. Chan School of Public Health, Harvard University, Boston, MA USA; 57 Department of Immunology and Infectious Diseases, Harvard T.H. Chan School of Public Health, Harvard University, Boston, MA USA; 58 University Medical Centre Hamburg-Eppendorf, University Cancer Centre Hamburg (UCCH), Hamburg, Germany; 59 Prevention and Cancer Control, Cancer Care Ontario, Toronto, ON Canada; 60 Dalla Lana School of Public Health, University of Toronto, Toronto, ON Canada; 61 Lunenfeld Tanenbaum Research Institute, Mount Sinai Hospital, University of Toronto, Toronto, ON Canada; 62 Department of Preventive Medicine, USC Norris Comprehensive Cancer Center, Keck School of Medicine, University of Southern California, Los Angeles, CA USA; 63 Division of Oncology, Department of Medicine, Stanford University, Stanford, CA USA; 64 Department of Medicine I, University Hospital Dresden, Technische Universität Dresden (TU Dresden), Dresden, Germany; 65 Centre for Epidemiology and Biostatistics, Melbourne School of Population and Global Health, The University of Melbourne, Melbourne, Victoria Australia; 66 Department of Epidemiology, School of Public Health and Institute of Health and Environment, Seoul National University, Seoul, South Korea; 67 Department of Biostatistics, University of Washington, Seattle, WA USA; 68 Division of Human Nutrition, Wageningen University and Research, Wageningen, The Netherlands; 69 Institute of Environmental Medicine, Karolinska Institutet, Stockholm, Sweden; 70 University of Hawaii Cancer Center, Honolulu, HI USA; 71 Department of Family Medicine, University of Virginia, Charlottesville, VA USA; 72 Department of Clinical Genetics, Karolinska University Hospital, Stockholm, Sweden; 73 Department of Molecular Medicine and Surgery, Karolinska Institutet, Stockholm, Sweden; 74 Department of Health Science Research, Mayo Clinic, Scottsdale, AZ USA; 75 Biomedicine Institute (IBIOMED), University of León, León, Spain; 76 Department of Clinical Sciences, Faculty of Medicine, University of Barcelona, Barcelona, Spain; 77 Department of Epidemiology, University of Washington, Seattle, WA USA; 78 Clinical Genetics Service, Department of Medicine, Memorial Sloan-Kettering Cancer Center, New York, USA; 79 Department of Medicine, Weill Cornell Medical College, New York, USA; 80 Program in MPE Molecular Pathological Epidemiology, Department of Pathology, Brigham and Women’s Hospital, Harvard Medical School, Boston, MA USA; 81 Department of Oncologic Pathology, Dana-Farber Cancer Institute, Boston, MA USA; 82 The Clinical Epidemiology Unit, Memorial University Medical School, Newfoundland, Canada; 83 Department of Public Health and Primary Care, University of Cambridge, Cambridge, UK; 84 Department of Community Medicine and Epidemiology, Lady Davis Carmel Medical Center, Haifa, Israel; 85 Ruth and Bruce Rappaport Faculty of Medicine, Technion-Israel Institute of Technology, Haifa, Israel; 86 Clalit National Cancer Control Center, Haifa, Israel; 87 Division of Research, Kaiser Permanente Northern California, Oakland, CA USA; 88 Department of General, Visceral, Vascular, and Transplantation Surgery, University Hospital Rostock, Rostock, Germany; 89 Department of Cancer Epidemiology, H. Lee Moffitt Cancer Center and Research Institute, Tampa, FL USA; 90 Department of Medicine and Epidemiology, University of Pittsburgh Medical Center, Pittsburgh, PA USA; 91 Department of Internal Medicine, University of Utah, Salt Lake City, UT USA; 92 Division of Laboratory Genetics, Department of Laboratory Medicine and Pathology, Mayo Clinic, Rochester, MN USA; 93 Huntsman Cancer Institute and Department of Population Health Sciences, University of Utah, Salt Lake City, UT USA; 94 Medical Faculty, University of Heidelberg, Heidelberg, Germany; 95 Discipline of Genetics, Memorial University of Newfoundland, St. John’s, Canada; 96 University of Southern California, Preventative Medicine, Los Angeles, CA USA; 97 Institute of Epidemiology, Helmholtz Zentrum Munich, Neuherberg, Germany

**Keywords:** Bilirubin, Cancer, Colorectal cancer, Anti-oxidants, Mendelian randomization analysis

## Abstract

**Background:**

Bilirubin, a byproduct of hemoglobin breakdown and purported anti-oxidant, is thought to be cancer preventive. We conducted complementary serological and Mendelian randomization (MR) analyses to investigate whether alterations in circulating levels of bilirubin are associated with risk of colorectal cancer (CRC). We decided a priori to perform analyses separately in men and women based on suggestive evidence that associations may differ by sex.

**Methods:**

In a case-control study nested in the European Prospective Investigation into Cancer and Nutrition (EPIC), pre-diagnostic unconjugated bilirubin (UCB, the main component of total bilirubin) concentrations were measured by high-performance liquid chromatography in plasma samples of 1386 CRC cases and their individually matched controls. Additionally, 115 single-nucleotide polymorphisms (SNPs) robustly associated (*P* < 5 × 10^−8^) with circulating total bilirubin were instrumented in a 2-sample MR to test for a potential causal effect of bilirubin on CRC risk in 52,775 CRC cases and 45,940 matched controls in the Genetics and Epidemiology of Colorectal Cancer Consortium (GECCO), the Colon Cancer Family Registry (CCFR), and the Colorectal Transdisciplinary (CORECT) study.

**Results:**

The associations between circulating UCB levels and CRC risk differed by sex (*P*_heterogeneity_ = 0.008). Among men, higher levels of UCB were positively associated with CRC risk (odds ratio [OR] = 1.19, 95% confidence interval [CI] = 1.04–1.36; per 1-SD increment of log-UCB). In women, an inverse association was observed (OR = 0.86 (0.76–0.97)). In the MR analysis of the main *UGT1A1* SNP (rs6431625), genetically predicted higher levels of total bilirubin were associated with a 7% increase in CRC risk in men (OR = 1.07 (1.02–1.12); *P* = 0.006; per 1-SD increment of total bilirubin), while there was no association in women (OR = 1.01 (0.96–1.06); *P* = 0.73). Raised bilirubin levels, predicted by instrumental variables excluding rs6431625, were suggestive of an inverse association with CRC in men, but not in women. These differences by sex did not reach formal statistical significance (*P*_heterogeneity_ ≥ 0.2).

**Conclusions:**

Additional insight into the relationship between circulating bilirubin and CRC is needed in order to conclude on a potential causal role of bilirubin in CRC development.

## Background

Globally, colorectal cancer (CRC) is the third most common cancer and the second leading cause of cancer-related death [1]. CRC is more frequent in men than in women, and its burden is expected to increase by 60% to more than 2.2 million new cancer cases and 1.1 million cancer deaths by 2030 [2].

Chronic inflammation is one of the hallmark characteristics of cancer, and inflammatory cells can also release reactive oxygen species, which trigger mutations in cancer cells [3]. Due to the inflammatory roots of CRC [4], it might be a candidate for prevention by anti-inflammatory and anti-oxidative agents. A compelling body of evidence from experimental and clinical studies has demonstrated that serum bilirubin, a byproduct of hemoglobin breakdown, has substantial anti-inflammatory and anti-oxidative properties [5–9]. Blood levels of total bilirubin are usually less than 17.1 μmol/L and consist primarily of unconjugated bilirubin (UCB) [10], which is also normally present in the gut and can cross gut cell membranes [11]. In vitro, UCB is the most active anti-oxidant part of total bilirubin [11–13]. The liver selectively removes UCB from the blood, and UCB is conjugated by a uridine diphosphoglucuronyltransferase (UGT1A1), after which it is transported to the bowel via the bile, where it is unconjugated by bacteria and excreted in the stool or reabsorbed [5–9]. Men usually have higher total bilirubin levels than women due to lower estrogen levels [5, 14] and a higher red blood cell turn-over [15, 16].

As the heme pathway plays an important role against oxidative stress, *UGT1A1* gene polymorphisms might be predictive of genetic pre-disposition to cancer [17]. Congenital underexpression of UGT1A1 causes mild chronic unconjugated hyperbilirubinemia, known as “Gilbert’s syndrome (GS),” and is associated with a polymorphism of the 5′ end of the *UGT1A1* gene promoter. The frequency of Gilbert’s polymorphism is 30–45%; however, phenotypic hyperbilirubinemia is estimated to be 5–10% in Caucasians [18–20].

Few epidemiological studies have investigated the association between circulating bilirubin levels and CRC risk with inconsistent findings [17, 21–26]. Notably, these previous studies only considered total bilirubin, were of limited size, and were cross-sectional or retrospective in design with one exception [22].

In this study, we analyzed pre-diagnostic circulating levels of UCB in relation to CRC development in the European Prospective Investigation into Cancer and Nutrition (EPIC). Additionally, we applied a complementary Mendelian randomization (MR) approach to investigate a potential causal relationship between genetically raised bilirubin levels and CRC in large international genetics consortia. We decided a priori to perform analyses separately in men and women because of the well-established sex differences in blood levels of bilirubin [10] and suggestive evidence that bilirubin CRC associations may differ between men and women [17, 23].

## Methods

### Study population and collection of blood samples and data

EPIC is a multi-center prospective cohort of 521,330 participants (~ 70% women, 25–70 years), recruited between 1992 and 2000, predominantly from the general population in 23 centers of 10 European countries (Sweden, Denmark, Norway, Germany, France, Greece, Italy, Spain, the UK, and the Netherlands) [27]. Around 80% of the participants donated a blood sample at recruitment, and plasma/serum samples were collected according to standardized procedures [27, 28] and stored at the International Agency for Research on Cancer (IARC, Lyon, France, at − 196 °C in liquid nitrogen), except in Denmark (nitrogen vapor, − 150 °C) and Sweden (− 80 °C freezers). At recruitment, participants completed standardized lifestyle and personal history questionnaires, had their diet assessed covering the previous 12 months using validated country/center-specific dietary questionnaires, and had height and weight (self-reported in the Oxford center and Norway, measured elsewhere) assessed [28].

### Cancer case ascertainment and selection

A detailed explanation of cancer case selection and ascertainment in EPIC has been published previously [29]. Briefly, incident cancer cases were identified through population cancer registries (Denmark, Italy except Naples, The Netherlands, Norway, Spain, Sweden, and UK; complete follow-up for cancer incidence ranging between December 2004 and 2008) or by active follow-up (France, Germany, Greece, and Naples; complete follow-up ranging between December 2006 and June 2010), consisting of a combination of methods including health insurance records, cancer and pathology registries, and active follow-up of study subjects and their next of kin. Cases were coded by anatomic location as colon and rectal cancer cases, identified according to the 10th revision of the International Classification of Diseases (ICD-10) and the second revision of the International Classification of Disease for Oncology (ICD-O-2). Proximal colon cancers included those within the cecum, appendix, ascending colon, hepatic flexure, transverse colon, and splenic flexure (C18.0-18.5). Distal colon cancers included those within the descending (C18.6) and sigmoid (C18.7) colon. Overlapping (C18.8) and unspecified (C18.9) lesions of the colon were grouped among all colon cancers only (C18.0-C18.9). Rectal cancers were defined as tumors occurring at the recto-sigmoid junction (C19) or rectum (C20). CRC is the combination of the colon and rectal cancer cases. Anal canal cancers (C21) were excluded.

Controls were selected by incidence density sampling from all cohort members alive and cancer-free at the time of matching to cases (1:1) by sex, age at blood collection, study center, time of day at blood collection, fasting status, menopausal status, and phase of menstrual cycle at blood collection.

A total of 1386 CRC cases (374 proximal colon, 412 distal colon, 80 overlapping proximal plus distal colon, and 520 rectal cancers) and 1386 controls were included in the current analyses.

### Laboratory measurement of circulating bilirubin

Circulating UCB levels were measured in plasma samples following a well-established protocol [30, 31] using high-performance liquid chromatography (HPLC, Merck, Hitachi, LaChrom, Vienna, Austria), equipped with a Fortis C18 HPLC-column (4.6 × 150 mm, 3 μm), a Phenomenex SecurityGuard™ cartridges for C18 HPLC-columns (4 × 3 mm), and a photodiode array detector (PDA, Shimadzu). An isocratic mobile phase contained glacial acetic acid (6.01 g/L) and 0.1 M n-dioctylamine in HPLC grade methanol/water (96.5/3.5%). Before starting the procedure, all aliquots were centrifuged and 50 μL plasma/serum was mixed with 200 μL mobile phase. After a second centrifugation, 120 μL of the supernatant was injected to the HPLC at a flow of 1 ml/min.

Case-control pairs were analyzed in the same plate to minimize batch-to-batch fluctuation. Bilirubin (alpha) (purity ≥ 98%, Sigma Aldrich) acted as an external standard (3.3% IIIα, 92.8% IXα, and 3.9% XIIIα isomers, 450 nm). One reference plasma sample was assessed per analysis as internal standard. The coefficient of variation (CV) between each plate was 6%.

### Genetic data

#### Genetic determinants for bilirubin levels

Genetic instruments for the MR analysis were identified as single-nucleotide polymorphisms (SNPs) associated with total bilirubin levels in the largest genome-wide association study (GWAS) (*P* < 5 × 10^−8^) conducted to date that included 317,639 individuals of European ancestry from the UK Biobank study [32]. UK Biobank is a prospective cohort that recruited more than 500,000 men and women aged 40–96 years between 2006 and 2010 and collected anthropometric, health, and lifestyle data and biological samples [33]. Explained phenotypic variance for a single SNP was estimated as a function of effect size for the risk factor in standard deviation units and minor allele frequency [34]. The strength of associations between the genetic instrument and bilirubin levels is reflected in the F-statistic, which is inversely related to weak instrument bias, being 10 the minimum estimation for a F-statistic to avoid bias of this nature [35]. The F-statistic was estimated as $F=\left(\frac{n-k-1}{k}\right)\left(\frac{R^2}{1-{R}^2}\right)$, where *R*^*2*^ is the proportion of phenotypic variance explained by the genetic instrument, *n* is the sample size, and *k* the number of genetic variants [35]. A total of 115 SNPs were identified as genetic instruments for total bilirubin, explaining 20.0% of phenotypic variance in circulating total bilirubin levels with an F-statistic of 696.5.

The SNP with the largest contribution was rs6431625 in the *UTG1A1* gene on chromosome 2. This SNP explained 16.9% of phenotypic variance and was in strong linkage disequilibrium (LD *R*^2^ = 0.74) with the UGT1A1*28 promoter TA repeat polymorphism (rs3064744) in European populations [36]. The other SNPs explained a 3.1% of phenotypic variance with an F-statistic of 89.1. All SNPs were independently associated with total bilirubin levels (LD *R*^2^ < 0.001), and SNPs with ambiguous strand codification (A/T or C/G) were replaced by SNPs in LD *R*^2^ > 0.8 in European populations using the *proxysnps* R package. As described in the GWAS where SNPs were identified, raw total bilirubin levels were adjusted for age, sex and their interaction, the top 40 principal components for population stratification, recruitment center, socioeconomic status, and potential technical confounders (blood draw time and its square and interactions with age and sex; urine sample time and its square and interactions with age and sex; sample dilution factor; fasting time, its square, and interactions with age and sex; and interactions of blood draw time and urine sample time with dilution factor) [32]. These adjusted residuals were inverse-normal-transformed and reflect the genetic association with bilirubin levels in standard deviation units (Supplementary Table 1, see Additional file 1). Total bilirubin is the sum of UCB (~ 80–85%) and conjugated bilirubin (~ 15–20%), and this ratio is constant under physiologic conditions.

#### Genome-wide data on CRC risk

Epidemiological and genetic data were derived from 51 studies (Supplementary Table 2, see Additional file 1) participating in the Genetics and Epidemiology of Colorectal Cancer Consortium (GECCO) [37], the Colon Cancer Family Registry (CCFR) [38], and the Colorectal Transdisciplinary (CORECT) study [39]. Men and women with incident invasive colorectal adenocarcinoma (ICD-9, codes 153-154) were included as cases. All CRC cases were confirmed by medical records, pathology reports, or death certificates. A total of 52,775 cases and 45,940 matched controls were included in the analyses [40]. On average, 51% of the study participants were men and the mean age was ~ 60 years; in all studies, controls were matched to cases on age and sex. The UK Biobank CRC cases and controls were excluded from the genetic consortia. This should prevent that weak instruments (i.e., genetic instruments not explaining much variation in circulating bilirubin) bias the MR risk estimate towards observed traditional risk estimates due to sample overlap between the SNP discovery sample (UK Biobank) and the CRC case-control samples [34].

Genotype information was available for all included studies. Details on genotyping, quality assurance, and imputation are described elsewhere [41]. In short, SNPs were excluded based on call rate (< 98% GECCO; < 95% CORECT), lack of Hardy-Weinberg equilibrium in controls (*P* < 1 × 10^−4^), or low minor allele frequency (≤ 1%). Analyses were restricted to individuals self-reported as of European descent and clustering with Utah residents with Northern/Western European ancestry from the CEU population in principal component analysis, including the HapMap II populations as reference. Summary statistics for genetic association with CRC risk were obtained for all studies included in the consortia and are shown in Supplementary Table 1.

### Statistical analyses

#### Serological analyses

Our a priori decision to perform all statistical analyses separately in men and women was confirmed by a strong effect modification by sex with regard to CRC risk in EPIC (*P*_heterogeneity_ = 0.008). Conditional logistic regression models were used to estimate odds ratios (OR) and 95% confidence intervals (CI) for associations between log-transformed UCB levels (log-UCB), standardized per one standard deviation (1-SD) increments, and CRC risk. Two models were constructed: a crude model which was conditioned on the matching criteria and then a multivariable model adjusted for level of education (none/primary school, technical/professional, secondary school, university degree), BMI (continuous, kg/m^2^), height (continuous), smoking status (never, former, and current smoker), physical activity (inactive, moderately inactive, moderately active, and active), alcohol consumption (g/day), dietary intakes of fiber (g/day), red meat (g/day), processed meats (g/day), dairy products (g/day), and total energy intake (kcal/day), and in women ever use of hormone therapy (HT, yes/no). Based on prior knowledge about the causal structure, we adjusted for variables that allowed all backdoor paths to be blocked in the directed acyclic graph (DAG) shown in Supplementary Figure 3, while avoiding adjustment for variables affected by either the exposure or the outcome [42]. Missing values in any of the categorical covariates were treated as a separate category.

We also investigated the potential non-linear dose-response associations between circulating levels of UCB and CRC risk. We used three-knot restricted cubic spline models at Harrell’s default percentiles (i.e., 10th, 50th, and 90th) in combination with a Wald-type test [43].

We tested for effect modification by categories of age (median), BMI (median), alcohol consumption (median), smoking status, menopausal status, use of HT, genotypes of the main *UGT1A1* SNP (rs6431625), and follow-up time (categories) by adding in the multivariable model a multiplicative interaction term between log-UCB and each of the aforementioned variables at a time. These hypothesis-free analyses were meant to assess the consistency of associations across population subgroups. Additional heterogeneity analysis was performed by cancer sub-sites (colon vs. rectum and proximal vs. distal). For this, we fitted stratified conditional logistic regression models based on competing risks and calculated the OR and their 95% CI in the subgroups of interest [44].

Finally, to evaluate the robustness of the results and address potential sources of bias such as reverse causation and residual confounding, we performed a range of sensitivity analyses. To exclude individuals with hepatic impairment, we calculated BTR index (the molar ratio of branched-chain amino acids to tyrosine) [45] and Fischer’s ratio (the molar ratio of branched-chain amino acids to tyrosine and phenylalanine) [46], which are clinical indicators of liver dysfunction and metabolism. Last, the fully adjusted models in EPIC were repeated after excluding subjects with missing values in any covariate.

To validate the genetic instruments for total bilirubin, we regressed the allele dose of the bilirubin-increasing allele of the main SNP (rs6431625) in the *UGT1A1* gene on the measured bilirubin levels in the EPIC sample with available GWAS data (*N* controls = 808).

#### Genetically predicted total bilirubin levels vs. CRC risk in GECCO/CCFR/ and CORECT

We investigated the genetic instruments for total bilirubin levels in relation to CRC risk using a 2-sample MR in 52,775 cases and 45,940 control participants within GECCO, CCFR, and CORECT (28,207 cases/22,204 controls in men and 24,568 cases/23,736 controls in women). With this sample size, the power was 80% to detect an OR ≥ 1.065 for the sex-stratified analyses per one standard deviation increment of total bilirubin levels.

Each genetic variant provides an estimation of the total bilirubin level effect on cancer risk (Wald estimate: genetic effect on CRC risk/genetic effect on total bilirubin levels). Before performing the main MR analysis, we assessed the presence of outlier observations within the SNP Wald estimates using the MR pleiotropy residual sum and outlier (MR-PRESSO) test [47]. This method identifies heterogeneity between SNP effects (*P*_Global_) as an evidence of horizontal pleiotropy, identifies outlier SNPs, and tests if the presence of outliers is biasing the estimation of risk (*P*_Distortion_). Then, as the main MR approach used in this study, SNP Wald estimates were combined in a single causal estimation through a likelihood-based MR approach, which is considered the most accurate MR method to estimate effects when there is a continuous log-linear association between risk factor and disease risk [48]. The multiplicative random effects inverse-variance weighted MR estimator was also applied [49]. However, the presence of pleiotropic variants can lead to biased causal effect estimates. In order to overcome this potential issue, several MR sensitivity analyses for data with potentially invalid instruments were applied. Initially, to evaluate the extent to which directional pleiotropy (non-balanced horizontal pleiotropy) may affect the effect estimate, we used the intercept test within an MR-Egger weighted linear regression approach [50]. Furthermore, two additional approaches, namely the weighted median method [51] and the modal-based estimate approach [52], relying on the distribution on SNP effects, were applied. In the former, the causal effect estimate is weighted towards the median of the distribution of SNPs used in the genetic instrument, while in the latter, the effect estimate is reflected by the mode of density distribution provided by SNP Wald estimates. Both methods are less sensitive to SNPs with biased effects. Finally, to identify whether the strongest SNP (rs6431625) was driving the association estimates, we obtained MR estimates leaving out this SNP from the SNP set.

Additionally, we investigated the between-sex heterogeneity of main causal effects by estimating the percentage of variance that is attributable to sex heterogeneity (*I*^2^ statistic), and the *P* value derived from Q statistic for heterogeneity (*P*_heterogeneity_), assuming a fixed-effect model of 1 degree of freedom.

Scatter plots were used to depict the genetic association on total bilirubin levels and CRC risk. All statistical analyses and plots were performed using Stata SE14 (Stata Corporation, College Station, TX, USA) and R (*MRPRESSO*, *TwoSampleMR*, and *ggplot2*; The R project). The significance testing was based on two-sided *P* values of less than 0.05.

## Results

Baseline characteristics of the EPIC participants are shown in Table 1. Mean follow-up time from blood collection to cancer diagnosis was 4.3 years (± 2.5 SD). Among men, cases compared to controls had higher UCB concentrations, were heavier (higher weight and BMI), and consumed more alcohol. Among women, cases compared to controls had lower UCB concentrations, were heavier (higher weight) and taller, and consumed less dairy products.

**Table 1:** Baseline characteristics of colorectal cancer cases and their matched controls by sex in the EPIC nested case-control study

Parameters	Men			Women		
	Case	Control	*P*	Case	Control	*P*
***N***	658	658		728	728	
**Age at blood collection (years)**	58.6 (7.1)	58.5 (7.1)	0.9	58.1 (7.7)	58.0 (7.7)	0.9
**Follow-up from blood collection (years)**	4.3 (2.5)			4.3 (2.5)		
**Weight (kg)**	82.5 (12.1)	80.4 (11.1)	0.001	68.5 (12.4)	66.8 (10.8)	0.007
**Height (cm)**	173.8 (6.8)	173.4 (6.8)	0.3	161.5 (6.5)	160.8 (6.5)	0.03
**BMI (kg/m**^**2**^**)**	27.3 (3.8)	26.7 (3.3)	0.01	26.3 (4.7)	25.9 (4.2)	0.08
**UCB (μmol/L)**	4.3 (2.6)	4.0 (2.2)	0.02	3.2 (1.8)	3.4 (1.9)	0.08
**Frequency of main SNP (rs6431625) (*n*, %)**			0.1			0.2
TT genotype (wild-type)	163 (36)	147 (41)		219 (39)	159 (35)	
TC genotype	216 (48)	160 (45)		258 (46)	218 (48)	
CC genotype	75 (17)	50 (14)		83 (15)	74 (16)	
**Smoking status (*n*, %)**			0.2			> 0.9
Never	156 (24)	185 (28)		426 (59)	428 (59)	
Former	310 (47)	278 (42)		160 (22)	159 (22)	
Current	180 (27)	184 (28)		138 (19)	137 (19)	
**Physical activity (*n*, %)**^**†**^			0.3			0.1
Inactive	157 (24)	155 (24)		207 (28)	170 (23)	
Moderately inactive	191 (29)	184 (28)		248 (34)	269 (37)	
Moderately active	159 (24)	137 (21)		154 (21)	144 (20)	
Active	143 (22)	169 (26)		116 (16)	140 (19)	
**Education (*n*, %)**^**‡**^			0.1			0.9
None	39 (6)	39 (6)		43 (6)	40 (5)	
Primary school completed	227 (35)	241 (37)		238 (33)	258 (35)	
Technical/professional school	154 (23)	177 (27)		170 (23)	166 (23)	
Secondary school	84 (13)	54 (8)		141 (19)	135 (19)	
Longer education (incl. university deg.)	139 (21)	131 (20)		104 (14)	111 (15)	
Unknown	9 (1)	12 (2)		27 (4)	15 (2)	
**Menopause stage (*n*, %)**						0.6
Pre-menopausal				85 (12)	90 (12)	
Post-menopausal				507 (70)	515 (71)	
Peri-menopausal				98 (13)	95 (13)	
Surgical postmen (bilateral ovariectomy)				38 (5)	28 (4)	
**Ever use of HT (*n*, %)**						0.8
No				533 (73)	526 (72)	
Yes				165 (23)	174 (24)	
**Daily dietary intake, median (5th, 95th percentile)**						
Energy (kcal)	2286 (1383, 3558)	2278 (1410, 3488)	0.8	1870 (1093, 2906)	1860 (1191, 2850)	0.7
Alcohol (g)	15 (0, 80)	13 (0, 71)	0.04	3 (0, 33)	4 (0, 33)	0.6
Red meat (g)	51 (8, 145)	49 (7, 135)	0.4	38 (4, 105)	40 (3, 105)	0.9
Processed meat (g)	34 (4, 111)	32 (2, 111)	0.1	21 (1, 71)	20 (1,68)	0.7
Fiber (g)	23 (12, 38)	23 (12, 40)	0.2	21 (12, 35)	22 (12, 34)	0.1
Dairy products (g)	257 (36, 765)	282 (43, 767)	0.1	299 (50, 801)	324 (63, 813)	0.02
**Country (*n*, %)**			> 0.9			> 0.9
France				40 (5)	40 (5)	
Italy	77 (12)	77 (12)		108 (15)	108 (15)	
Spain	86 (13)	86 (13)		79 (11)	79 (11)	
UK	123 (19)	123 (19)		125 (17)	125 (17)	
The Netherlands	23 (3)	23 (3)		147 (20)	147 (20)	
Greece	21 (3)	21 (3)		19 (3)	19 (3)	
Germany	120 (18)	120 (18)		64 (9)	64 (9)	
Sweden	44 (7)	44 (7)		30 (4)	30 (4)	
Denmark	164 (25)	164 (25)		105 (14)	105 (14)	
Norway				11 (2)	11 (2)	
**Fasting status (*n*, %)**			> 0.9			> 0.9
No	324 (50)	324 (50)		361 (51)	360 (51)	
Inbetween	141 (22)	140 (22)		139 (19)	139 (19)	
Yes	185 (28)	185 (28)		215 (30)	215 (30)	

Values are means (SD) unless stated otherwise. Categorical variables are expressed as *n* (%) and continuous variables as means (SD) or medians (5, 95%). Paired *T* test (mean comparison) or Wilcoxon rank sum test for dietary intakes and chi-square test for categorical variables were used to calculate the *P* value. Number of missing values (cases/controls): physical activity (12/18), smoking status (16/15), education (11/7), and HT (30/28). Missing values were not excluded in percentage calculations; therefore, the percent’s sum across subgroups is not 100%

*Abbreviations*: *N* number, *UCB* unconjugated bilirubin, *BMI* body mass index, *HT* hormone therapy

^†^A study participant was considered active if he/she reported a leisure time activity of at least 1 h per week in at least one season

^‡^Education level was defined as high in case of final secondary school examination and otherwise as low. More details have been published previously [27, 28]

There was a suggestive higher frequency of genotypes in the homozygotes or heterozygotes than the frequency of the wild-type in CRC cases as compared to controls in men, and less so in women.

### Serological analyses: association between circulating bilirubin levels and CRC risk

In the EPIC cohort, among men, we observed a positive association between pre-diagnostic UCB levels and CRC risk in both crude and multivariable adjusted models (multivariable OR = 1.19, 95% CI = 1.04–1.36; *P* = 0.01; per 1-SD increment in log-UCB). In contrast, we observed an inverse association between UCB and CRC risk in women in both crude and multivariable adjusted models (multivariable OR = 0.86, 95% CI = 0.76–0.97; *P* = 0.02; per 1-SD log-UCB increment) (Table 2). These associations followed a linear trend in men (*P*_nonlinearity_ = 0.7) and in women (*P*_nonlinearity_ = 0.1), but for the latter with little change in the OR between 4 to 15 μmol/L of UCB (Supplementary Figure 1, see Additional file 1).

**Table 2:** Odds ratio and 95% confidence interval for the association between bilirubin levels and CRC risk

	Men			Women		
		Odds ratio (95% CI)			Odds ratio (95% CI)	
	*n* cases/controls	Crude	Adjusted	*n* cases/controls	Crude	Adjusted
**Nested case-control study EPIC (1-SD)**^†^						
log-UCB	658/658	1.13 (1.00–1.28)	1.19 (1.04–1.36)	728/728	0.86 (0.77–0.97)	0.86 (0.76–0.97)
*P*		0.05	0.01		0.01	0.02
MR approach for total bilirubin (1-SD)*						
rs6431625 Wald estimate^§^	28,270/22,204		1.07 (1.02–1.12)	24,568/23,736		1.01 (0.96–1.06)
*P*			0.006			0.73
114 SNPs likelihood-based MR estimate^§^	28,270/22,204		0.89 (0.80–1.00)	24,568/23,736		1.00 (0.89–1.11)
*P*			0.05			0.96

*Abbreviations*: *n* number, *P P* value, *CI* confidence interval, *log-UCB* log-transformed unconjugated bilirubin

^†^EPIC (European Prospective Investigation into Cancer and Nutrition): Conditional logistic regression models were used to estimate odds ratios (OR) and 95% confidence intervals (CI) for associations between log-transformed UCB levels (log-UCB), standardized per one standard deviation (1-SD) increments, and CRC risk. The crude model was conditioned on the matching factors including study center, age at blood collection (1 year), fasting status and time (3 h intervals) at blood collection, among women, additionally by menopausal status (pre-, peri-, and post-menopausal or surgically menopausal), and hormone therapy (HT) (yes, no). The multivariable model was adjusted for level of education (none/primary school, technical/professional, secondary school, university degree), BMI, height, smoking status (never, former, current smoker), physical activity (inactive, moderately inactive, moderately active, active), alcohol consumption (g/day), dietary intakes of fiber (g/day), red meat (g/day), processed meats (g/day), dairy products (g/day), and total energy intake (kcal/day)

*MR approach: Mendelian randomization approach; data from the Colon Cancer Familiar Registry (CCFR), the Colorectal Transdisciplinary (CORECT) study, and the Genetics and Epidemiology of Colorectal Cancer Consortium (GECCO)

^§^Odds ratio and 95% confidence interval for colorectal cancer per 1-SD increment in bilirubin levels estimated through a likelihood-based MR approach

### Effect modification and sensitivity analyses

The association of UCB levels with CRC risk in EPIC women differed by age (Table 3). We observed an inverse association between UCB levels and CRC risk in older women (> 58.5 years) (multivariable OR = 0.73, 95% CI = 0.61–0.87; *P* = 0.001; per 1-SD increment in log-UCB), but not in younger women (OR = 1.01, 95% CI = 0.85–1.19; *P >* 0.9) (*P*_heterogeneity_ = 0.008). Serum levels of UCB were lower in older women compared with younger women. No effect modification by age at blood collection was observed in men (*P*_heterogeneity_ = 0.3).

**Table 3:** The association between unconjugated bilirubin (UCB) levels and colorectal cancer risk across strata of potential effect modifiers in the EPIC study

Colorectal cancer								
Variables	Men				Women			
	*n* cases/controls^†^	Odds ratio (95% CI)	*P*	*P*_heterogeneity_	*n* cases/controls^†^	Odds ratio (95% CI)	*P*	*P*_heterogeneity_
Adjusted model	**658/658**	**1.19 (1.04–1.36)**	**0.01**		**728/728**	**0.86 (0.76–0.97)**	**0.02**	
Age at blood collection (year, median)	658/658			0.30	728/728			**0.008**
**< 59.2/< 58.5**	329/329	1.28 (1.06–1.54)			364/364	1.01 (0.85–1.19)	> 0.90	
**≥ 59.2/≥ 58.5**	329/329	1.10 (0.92–1.32)			364/364	0.73 (0.61–0.87)	0.001	
rs6431625 (increasing levels C allele)	333/333			**0.02**	428/428			0.14
**TT genotype (wild-type)**	117/139	1.1 (0.73–1.65)	0.60		168/151	0.69 (0.49–0.99)	0.04	
**TC genotype**	160/146	0.92 (0.64–1.32)	0.60		194/206	0.71 (0.53–0.95)	0.02	
**CC genotype**	56/48	2.01 (1.26–3.20)	0.003		66/71	1.06 (0.74–1.53)	0.7	
Smoking status	658/658			0.40	728/728			0.20
Never	170/170	1.26 (1.00–1.59)	0.05		427/427	0.83 (0.71–0.98)	0.03	
Former	294/294	1.06 (0.86–1.30)	0.60		160/160	0.84 (0.66–1.07)	0.20	
Current	182/182	1.33 (1.08–1.64)	0.01		137/137	0.99 (0.78–1.25)	0.90	
Unknown	12/12	0.93 (0.35–2.49)	0.90		4/4	0.05 (0.00–0.1)	0.20	
Follow-up time (years)	658/658			0.20	728/728			0.60
1 (< 2)	141/141	0.96 (0.74–1.25)	0.80		158/158	0.82 (0.65–1.05)	0.10	
2 (2–4)	173/173	1.34 (1.04–1.72)	0.02		160/160	0.96 (0.76–1.23)	0.80	
3 (> 4)	244/244	1.23 (1.03–1.50)	0.02		280/380	0.84 (0.71–0.99)	0.04	

No effect modifications by BMI (median), alcohol consumption (median), menopausal status, and use of HT were observed (all *P* ≥ 0.7)

*Abbreviations*: *n* number, *P P* value, *CI* confidence interval

^†^Cases matched 1:1 to control subjects. EPIC (European Prospective Investigation into Cancer and Nutrition): Conditional logistic regression models were used to estimate odds ratios (OR) and 95% confidence intervals (CI) for associations between log-transformed UCB levels (log-UCB), standardized per one standard deviation (1-SD) increments, and CRC risk. The crude model was conditioned on the matching factors including study center, age at blood collection (1 year), fasting status and time (3 h intervals) at blood collection, among women, additionally by menopausal status (pre-, peri-, and post-menopausal or surgically menopausal), and hormone therapy (HT) (yes, no). The multivariable model was adjusted for level of education (none/primary school, technical/professional, secondary school, university degree), BMI, height, smoking status (never, former, current smoker), physical activity (inactive, moderately inactive, moderately active, active), alcohol consumption (g/day), dietary intakes of fiber (g/day), red meat (g/day), processed meats (g/day), dairy products (g/day), and total energy intake (kcal/day)

In contrast, in men (*P*_heterogeneity_ = 0.02), but not in women (*P*_heterogeneity_ = 0.14), effect modification by the rs6431625 genotype was observed (Table 3). In men with homozygous genotype of the bilirubin-increasing effect allele (CC) in rs6431625, higher levels of measured UCB were positively associated with CRC risk (OR = 2.01, 95% CI = 1.26–3.20; *P* = 0.003; per 1-SD increment in log-UCB), while no associations were observed in those with heterozygous (TC) or wild-type (TT) genotypes. Homozygote *UGT1A1* bilirubin-increasing allele carriers (rs6431625) had higher serum UCB levels compared to heterozygotes or wild-type in the EPIC population with GWAS data (*R*^2^ = 0.20; *P* < 0.001, *N* controls = 808) (Supplementary Figure 2, see Additional file 1).

No differences in the association between UCB levels and CRC risk in men and women were observed across categories of BMI, alcohol consumption, smoking status, menopausal status, use of HT, and follow-up time in years (Table 3). Estimated associations between UCB levels and CRC risk in men and women were also robust to sensitivity analyses (Supplementary Table 3, see Additional file 1).

There was no heterogeneity in associations by anatomical sub-sites (colon vs. rectum, or proximal colon vs. distal colon) (all *P*_heterogeneity_ ≥ 0.1) (Table 4).

**Table 4:** The association between unconjugated bilirubin (UCB) concentrations and colorectal cancer risk by anatomical sub-sites in the EPIC study

Colorectal cancer						
	Men			Women		
	*n* cases/controls^†^	Odds ratio (95% CI)	*P*	*n* cases/controls^†^	Odds ratio (95% CI)	*P*
Adjusted model	**658/658**	**1.19 (1.04–1.36)**	**0.01**	**728/728**	**0.86 (0.76–0.97)**	**0.02**
Anatomical site	658/658		> 0.9^‡^	728/728		0.2^‡^
Colon	381/381	1.18 (0.99–1.42)	0.07	485/485	0.81 (0.70–0.95)	0.008
Rectum	277/277	1.19 (0.99–1.43)	0.06	243/243	0.95 (0.62–0.79)	0.79
Colon sub-site	339/339		0.1^‡^	447/447		0.9^‡^
Proximal	156/156	1.10 (0.83–1.47)	0.5	218/218	0.77 (0.62–0.95)	0.017
Distal	183/183	1.55 (1.15–2.11)	0.01	229/229	0.79 (0.62–1.00)	0.06

EPIC (European Prospective Investigation into Cancer and Nutrition): Conditional logistic regression models were used to estimate odds ratios (OR) and 95% confidence intervals (CI) for associations between log-transformed UCB levels (log-UCB), standardized per one standard deviation (1-SD) increments, and CRC risk. The crude model was conditioned on the matching factors including study center, age at blood collection (1 year), fasting status and time (3 h intervals) at blood collection, among women, additionally by menopausal status (pre-, peri-, and post-menopausal or surgically menopausal), and hormone therapy (HT) (yes, no). The multivariable model was adjusted for level of education (none/primary school, technical/professional, secondary school, and university degree), BMI, height, smoking status (never, former, current smoker), physical activity (inactive, moderately inactive, moderately active, and active), alcohol consumption (g/day), dietary intakes of fiber (g/day), red meat (g/day), processed meats (g/day), dairy products (g/d), and total energy intake (kcal/day)

*Abbreviations*: *n* number, *P P* value, *CI* confidence interval

^†^Cases matched 1:1 to control subjects

^‡^*P*_heterogeneity_

### Genetically predicted bilirubin levels and CRC risk in GECCO/CCFR/ and CORECT

In light of the heterogeneous results across the main *UGT1A1* SNP (rs6431625) genotype categories in serological analyses, we applied a MR approach to this SNP separately from the other genetic instruments. In the MR analysis of the rs6431625, higher levels of genetically predicted bilirubin were positively associated with CRC risk in men (OR = 1.07, 95% CI = 1.02–1.12; *P* = 0.006; per 1-SD of total bilirubin), but not in women (OR = 1.01, 95% CI = 0.96–1.06; *P* = 0.73) (Table 2) (*I*^2^ = 64.0%; *P*_heterogeneity_ = 0.10).

**Fig. 1:**
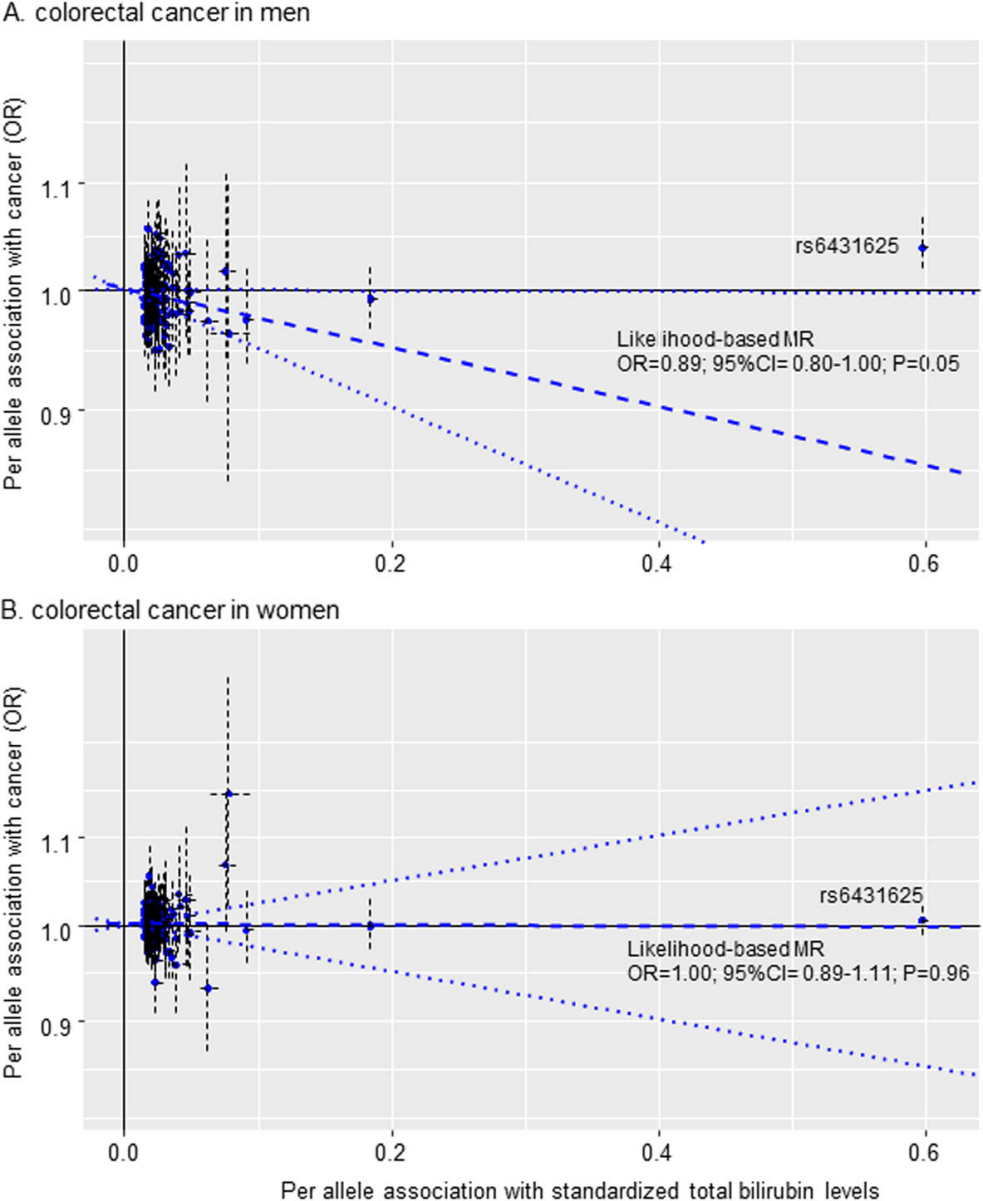
Scatter plots depicting the genetic association between total bilirubin levels and colorectal cancer risk. Per allele association of total bilirubin SNPs with inverse-normal-transformed bilirubin levels (*x* axis) and risk for colorectal cancer (*y* axis; logarithmic scale) in men (**a**) and in women (**b**), together with the likelihood-based MR estimate for the genetic instrument comprising of the 114 SNPs (dashed-blue line) and their 95% CI (dotted-blue lines)

In the MR analyses of the other 114 genetic instruments, no outlier SNPs were identified by MR-PRESSO analyses, with some heterogeneity among the instruments (*P*_Global_ < 0.04). The likelihood-based MR risk estimates, which excluded rs6431625, showed some evidence that higher levels of bilirubin were inversely associated with CRC risk in men (OR = 0.89, 95% CI = 0.80–1.00; *P* = 0.05), while in women, no association was observed (OR = 1.00, 95% CI = 0.89–1.11; *P* = 0.96) (Table 2) (*I*^2^ = 39.0%; *P*_heterogeneity_ = 0.20). Scatter plots depicting the genetic association of the 115 SNPs with total bilirubin levels and with CRC risk, together with MR risk estimates for the genetic instrument comprising the 114 SNPs, are shown in Fig. 1.

In MR sensitivity analyses of the 114 SNP instrument, the MR-Egger test did not detect directional pleiotropy in the intercept analysis for total bilirubin levels in men or women (*P*_intercept_ ≥ 0.45). The additional inverse-variance weighting, weighted median, and modal-based estimates provided similar results compared to the likelihood-based MR risk estimates (Supplementary Table 4, see Additional file 1).

## Discussion

We investigated the relation between pre-diagnostic levels of circulating UCB, the main component of total bilirubin, and CRC risk in the EPIC study, and then complemented these analyses with an MR approach using data from large-scale genetic consortia of CRC. In the serological analysis, higher circulating levels of UCB were positively associated with CRC risk in men and inversely associated in women. The complementary MR analysis supported a positive association between total bilirubin levels, genetically predicted by a *UGT1A1* SNP (rs6431625), and CRC risk in men, but not in women. We further found that bilirubin levels predicted by instrumental variables excluding the *UGT1A1* SNP were suggestive of an inverse association with CRC in men, which is in line with our initial hypothesis, but not in women.

These directionally different associations of bilirubin-raising genetic instruments with CRC in men suggest that the *UGT1A1* SNP either has horizontal pleiotropic effects through pathways other than elevated blood levels of bilirubin or indicates an elevated bilirubin distribution among individuals with GS as compared to the general population. Both scenarios are biologically plausible.

Potential pleiotropic effects of the *UGT1A1* SNP include a reduced capacity of the UGT1A1 enzyme in the liver or gut to metabolize xenobiotics and toxic substances (e.g., heterocyclic aromatic amines, in well-done red meat) [24]. Furthermore, the influence of sex hormones on UGT1A1 activity [14], and differences in *UGT1A1* expression between men and women, leading to differential bilirubin conjugation and circulating levels [53], might partly explain the sex differences in CRC risk found in this study. There is suggestive evidence for sex differences in the *UGT1A1* variants and CRC risk [23]. In our control outcome and yet unpublished work, we observed similar sex differences in associations between bilirubin, predicted by the same *UGT1A1* SNP, and risk of pancreatic cancer (suggestive positive association in men and null association in women) using data of genetic consortia on pancreatic cancer (Supplementary Table 5, see Additional file 1).

In a second scenario, the findings in men could indicate that bilirubin, an anti-oxidant in vitro [30, 54–56], could trigger pro-oxidative processes at high-normal levels in the gut, similar to what has been described for ascorbic acid [57]. Both serological and MR analyses indicated that increased CRC risk was confined to men with a genetic pre-disposition to high bilirubin levels (in our study: bilirubin effect allele (CC) in rs6431625). It is estimated that 11–16% of Caucasians carry a homozygous bilirubin-increasing risk allele [58], and if one in ten individuals have a physiologic trait that affects their risk of cancer, this would have significant implications for future cancer prevention. Nevertheless, follow-up studies are needed to fully clarify the role of bilirubin in CRC development; for example, by conducting a multivariable MR [59], where bilirubin is jointly instrumented with potential other phenotype(s) that could be associated with *UGT1A1* variants.

The few studies to date that have investigated the association between circulating bilirubin levels and CRC risk have reported inconsistent results [17, 19, 22]. In an exploratory retrospective case-control study (174 cases), lower total bilirubin levels were associated with higher risk of CRC in men and in women [17]. In a prospective investigation in the National Health and Nutrition Examination Survey (NHANES I), a null association between total bilirubin levels and incidence of CRC was reported (110 cases men and women combined) [22], whereas a prior cross-sectional analysis in the NHANES III reported an inverse association between total bilirubin levels and CRC (83 cases, men and women combined) [19]. These inconsistencies are most likely attributable to differences in study design and/or limited sample sizes. The current analysis goes beyond previous studies in that we used a prospective design with pre-diagnostic blood samples and a large number of incident cases that provided sufficient power for sex stratification.

To our knowledge, no other studies to date have investigated potential causal association between circulating bilirubin and CRC risk using an MR approach. However, variants in the *UGT1A1* gene have been previously examined in relation to CRC. Consistent with our findings, a positive association between the UGT1A1*28 allele (homo-/heterozygous for higher bilirubin) and CRC risk in men (OR = 1.97, 95% CI = 1.22–3.19; *P* = 0.005), but not in women (*P* = 0.26) was reported in a Macedonian retrospective case-control study [23]. However, another retrospective case-control study [60], which combined men and women, found no significant association between UGT1A1*28 and CRC risk (OR = 1.10, 95% CI = 0.84–1.50). In contrast, Jiraskova et al. [17] reported an inverse association between the UGT1A1*28 polymorphism and CRC risk in men (OR = 0.75, 95% CI = 0.58–0.96) and also a non-significant inverse association in women (OR = 0.88, 95% CI = 0.66–1.18), which however may have limited generalizability due to a highly selected study sample. Our approach goes beyond these studies in terms of sample size, comprehensive SNP analyses and linking for the first time circulating bilirubin to a cancer outcome using an MR approach.

In subgroup analyses of our EPIC study, we found a stronger inverse association between UCB and CRC risk in older women (> 58.5 years) compared to younger women. This effect modification by age was not observed in men. The age patterns seen with bilirubin were observed in previous studies in respect to indicators of metabolic health in men and women [61, 62]. However, a more likely explanation for this finding in women is bias due to differential selection of women less susceptible for CRC over time [63].

The main strengths of our study were the prospective design with long follow-up time between blood sampling and CRC diagnosis, and large sample size to stratify by sex and anatomical sub-sites of CRC with access to biomarkers and lifestyle factors for a better control of potential confounding. Second, we applied an MR approach to address potential confounding, including residual confounding, and reverse causation in our serological analysis.

Our study was limited by the lack of liver enzyme data at baseline in the EPIC study to infer hepatic pathology which would impact bilirubin synthesis. In order to overcome this issue, we used Fischer’s ratio and BTR index for excluding those subjects potentially having liver abnormalities; therefore, we could be sure that participants who had higher UCB did not suffer from liver disease. Second, storage of samples for prolonged periods of time could have contributed to a degradation of UCB concentrations. As with traditional epidemiological analysis, selection bias can also adversely affect MR studies [63]. Given that attrition rates in the genetic consortia were reported as low [38, 39] and that the GWAS on bilirubin was not conditioned on another [32], selection bias may not explain our findings [64]. A major assumption in our MR was that the genetic instruments affect CRC risk only through bilirubin levels. Potential pleiotropic effects of our *UGT1A1* SNP (rs6431625) cannot be excluded, and pathways other than mild hyperbilirubinemia associated with lower UGT1A1 activity could therefore also play a role in CRC development [24]. Nevertheless, it is also biologically plausible that our observed associations reflect the effect of an elevated distribution of circulating bilirubin. This is supported by our serological finding that the positive association between serum levels of bilirubin and CRC risk was confined to men with a genetic pre-disposition to high bilirubin levels (in our study: bilirubin effect allele (CC) in rs6431625). A look-up at the PhenoScanner database indicated self-reported liver or biliary/pancreas problems, which likely hints at undiagnosed GS.

We also assessed potential horizontal pleiotropy of the other genetic instruments without the *UGT1A1* SNP [65]. The corresponding MR analysis after strictly removing all SNPs, (including those associated with yet unknown phenotypes), which might have violated the exclusion restriction (horizontal pleiotropy) and the independence assumption (no confounders) [59, 66], resulted in virtually similar associations, despite our conservative unsupervised approach (Supplementary Table 4). These excluded SNPs were genome-wide associated with educational attainment, BMI, mean corpuscular volume of red blood cells, and others (Supplementary Table 6). We also employed a set of sensitivity MR methods (e.g., conservative MR-Egger approach) [50], known to be robust for different types of pleiotropy, and there was no indication of horizontal pleiotropy in our MR analysis. Lastly, weak instruments in a two-sample MR study can bias estimates towards the null [51], which we deem unlikely in our study given the F-statistics of our *UGT1A1* SNP (*F* = 696.5) and of our other instruments (*F* = 89.1).

## Conclusions

In conclusion, we observed that higher circulating bilirubin levels were positively associated with CRC risk in men. Both serological and MR analysis suggested that increased CRC risk was confined to men with a genetic pre-disposition to high bilirubin levels. In women, the inverse relationship between circulating bilirubin and CRC risk observed in the serological analysis was not supported in the MR approach. Additional insight into the relationship between circulating bilirubin and CRC is needed in order to conclude on a potential causal role of bilirubin in CRC development.

## Supplementary information

**Additional file 1 Supplementary tables 1–6**, **supplementary figures 1–3**, **supplementary text 1**. **Suppl. table 1** - Summary statistics for the genetic association with bilirubin levels, and sex-stratified colorectal cancer risk. **Suppl. table 2** - Participating studies in the genetic consortia (GECCO/ CCFR/ and CORECT). **Suppl. table 3** - Associations between unconjugated bilirubin (UCB) levels and colorectal cancer risk after different sensitivity analyses in the EPIC study. **Suppl. table 4** - Results for the Mendelian randomization sensitivity analyses. **Suppl. table 5** - Results for the Mendelian randomization sensitivity analyses: positive control outcomes for pancreatic cancer. **Suppl. table 6** - Results for genome-wide associations of instruments with other phenotypes. **Suppl. figure 1** - Cubic spline modeling of unconjugated bilirubin (UCB) levels in relation to colorectal cancer risk in the EPIC study. **Suppl. figure 2** - Association between *UGT1A1* polymorphism (rs6431625) and unconjugated bilirubin (UCB) levels in the EPIC study (with available GWAS data). **Suppl. figure 3** - Directed acyclic graph (DAG) of the causal structure of associations between unconjugated bilirubin (UCB) levels in relation to colorectal cancer risk in the EPIC study. **Suppl. text 1** - Specific funding sources and acknowledgements of participating studies.

## Data Availability

For information on how to submit an application for gaining access to EPIC data and/or biospecimens, please follow the instructions http://epic.iarc.fr/access/index.php. For gaining access to GECCO/CCFR/CORECT, please see at https://www.fredhutch.org/en/labs/phs/projects/cancer-prevention/projects/gecco.html.

## Abbreviations

BMI: Body mass index
BTR: Branched-chain amino acids to tyrosine ratio
CCFR: Colon Cancer Familiar Registry
CORECT: Colorectal Cancer Transdisciplinary Research Study
CRC: Colorectal cancer
EPIC: European Prospective Investigation into Cancer and Nutrition
GECCO: Genetics and Epidemiology of Colorectal Cancer Consortium
GS: Gilbert’s syndrome
GWAS: Genome-wide association studies
HPLC: High-performance liquid chromatography
HT: Hormone therapy
IGF1: Insulin-like growth factor 1
IGF-BP: Insulin-like growth factor binding proteins
MR: Mendelian randomization
NHANES: Third National Health and Nutrition Examination Survey
SNP: Single-nucleotide polymorphism
TA: Thymine-adenine
UCB: Unconjugated bilirubin
UGT1A1: Uridine diphosphoglucuronate glucuronosyltransferase1A1

## References

[1] Bray F, Ferlay J, Soerjomataram I, Siegel RL, Torre LA, Jemal A (2018). Global cancer statistics 2018: GLOBOCAN estimates of incidence and mortality worldwide for 36 cancers in 185 countries. CA Cancer J Clin.

[2] Arnold M, Sierra MS, Laversanne M, Soerjomataram I, Jemal A, Bray F (2017). Global patterns and trends in colorectal cancer incidence and mortality. Gut..

[3] Hanahan D, Weinberg RA (2011). Hallmarks of cancer: the next generation. Cell..

[4] Lasry A, Zinger A, Ben-Neriah Y (2016). Inflammatory networks underlying colorectal cancer. Nat Immunol.

[5] Wagner KH, Wallner M, Molzer C, Gazzin S, Bulmer AC, Tiribelli C (2015). Looking to the horizon: the role of bilirubin in the development and prevention of age-related chronic diseases. Clin Sci (London).

[6] Sedlak TW, Saleh M, Higginson DS, Paul BD, Juluri KR, Snyder SH (2009). Bilirubin and glutathione have complementary antioxidant and cytoprotective roles. Proc Natl Acad Sci U S A.

[7] Rodrigues CM, Sola S, Brito MA, Brites D, Moura JJ (2002). Bilirubin directly disrupts membrane lipid polarity and fluidity, protein order, and redox status in rat mitochondria. J Hepatol.

[8] Hansen TW, Mathiesen SB, Walaas SI (1996). Bilirubin has widespread inhibitory effects on protein phosphorylation. Pediatr Res.

[9] Fevery J (2008). Bilirubin in clinical practice: a review. Liver int.

[10] Wagner KH, Shiels RG, Lang CA, Seyed Khoei N, Bulmer AC (2018). Diagnostic criteria and contributors to Gilbert’s syndrome. Crit Rev Clin Lab Sci.

[11] Zucker SD, Goessling W, Hoppin AG (1999). Unconjugated bilirubin exhibits spontaneous diffusion through model lipid bilayers and native hepatocyte membranes. J Biol Chem.

[12] Keshavan P, Schwemberger SJ, Smith DL, Babcock GF, Zucker SD (2004). Unconjugated bilirubin induces apoptosis in colon cancer cells by triggering mitochondrial depolarization. Int J Cancer.

[13] Corich L, Aranda A, Carrassa L, Bellarosa C, Ostrow JD, Tiribelli C (2009). The cytotoxic effect of unconjugated bilirubin in human neuroblastoma SH-SY5Y cells is modulated by the expression level of MRP1 but not MDR1. Biochem J.

[14] Muraca M, Fevery J (1984). Influence of sex and sex steroids on bilirubin uridine diphosphate-glucuronosyltransferase activity of rat liver. Gastroenterology..

[15] Molzer C, Wallner M, Kern C, Tosevska A, Zadnikar R, Doberer D (2017). Characteristics of the heme catabolic pathway in mild unconjugated hyperbilirubinemia and their associations with inflammation and disease prevention. Sci Rep.

[16] Murphy WG (2014). The sex difference in haemoglobin levels in adults - mechanisms, causes, and consequences. Blood Rev.

[17] Jiraskova A, Novotny J, Novotny L, Vodicka P, Pardini B, Naccarati A (2012). Association of serum bilirubin and promoter variations in HMOX1 and UGT1A1 genes with sporadic colorectal cancer. Int J Cancer.

[18] Zaman S, Fukushima H, Suzuki R, Hawlader M, Yoshimatsu S, Kanai UA, GU (2018). Prevalence of Gilbert syndrome in Bangladesh. Open J Blood Dis.

[19] Zucker SD, Horn PS, Sherman KE (2004). Serum bilirubin levels in the U.S. population: gender effect and inverse correlation with colorectal cancer. Hepatology (Baltimore).

[20] Ye J, Cui L, Zhou Y, Huang Y, Banafa O, Hou X (2018). “Gilbert’s-like” syndrome as part of a spectrum of persistent unconjugated hyperbilirubinemia in post-chronic hepatitis patients. Sci Rep.

[21] Tang KS, Chiu HF, Chen HH, Eng HL, Tsai CJ, Teng HC (2005). Link between colorectal cancer and polymorphisms in the uridine-diphosphoglucuronosyltransferase 1A7 and 1A1 genes. World J Gastroenterol.

[22] Ioannou GN, Liou IW, Weiss NS (2006). Serum bilirubin and colorectal cancer risk: a population-based cohort study. Aliment Pharmacol Ther.

[23] Bajro MH, Josifovski T, Panovski M, Jankulovski N, Nestorovska AK, Matevska N (2012). Promoter length polymorphism in UGT1A1 and the risk of sporadic colorectal cancer. Cancer Genet.

[24] Girard H, Butler LM, Villeneuve L, Millikan RC, Sinha R, Sandler RS (2008). UGT1A1 and UGT1A9 functional variants, meat intake, and colon cancer, among Caucasians and African-Americans. Mutat Res.

[25] Temme EH, Zhang J, Schouten EG, Kesteloot H (2001). Serum bilirubin and 10-year mortality risk in a Belgian population. Cancer Causes Control.

[26] Kuhn T, Sookthai D, Graf ME, Schubel R, Freisling H, Johnson T (2017). Albumin, bilirubin, uric acid and cancer risk: results from a prospective population-based study. Br J Cancer.

[27] Riboli E, Kaaks R (1997). The EPIC Project: rationale and study design. European Prospective Investigation into Cancer and Nutrition. In J Epidemiol.

[28] Riboli E, Hunt KJ, Slimani N, Ferrari P, Norat T, Fahey M (2002). European Prospective Investigation into Cancer and Nutrition (EPIC): study populations and data collection. Public Health Nutr.

[29] Jenab M, Bueno-de-Mesquita HB, Ferrari P, van Duijnhoven FJ, Norat T, Pischon T (2010). Association between pre-diagnostic circulating vitamin D concentration and risk of colorectal cancer in European populations:a nested case-control study. BMJ.

[30] Molzer C, Huber H, Steyrer A, Ziesel G, Ertl A, Plavotic A (2012). In vitro antioxidant capacity and antigenotoxic properties of protoporphyrin and structurally related tetrapyrroles. Free Radic Res.

[31] Wallner M, Bulmer AC, Molzer C, Mullner E, Marculescu R, Doberer D (2013). Haem catabolism: a novel modulator of inflammation in Gilbert’s syndrome. Eur J Clin Investig.

[32] Sinnott-Armstrong N TY, Amar D, Mars N, Aguirre M, Venkataraman GR, et al. Genetics of 38 blood and urine biomarkers in the UK Biobank. bioRxiv 660506; 2019. 10.1101/660506.

[33] Collins R (2012). What makes UK Biobank special?. Lancet (London).

[34] Burgess S, Davies NM, Thompson SG (2016). Bias due to participant overlap in two-sample Mendelian randomization. Genet Epidemiol.

[35] Burgess S, Thompson SG (2011). Avoiding bias from weak instruments in Mendelian randomization studies. Int J Epidemiol.

[36] National Cancer Institute. https://ldlink.nci.nih.gov/?tab=ldpair. [Accessed Jan 2020].

[37] Peters U, Jiao S, Schumacher FR, Hutter CM, Aragaki AK, Baron JA (2013). Identification of genetic susceptibility loci for colorectal tumors in a genome-wide meta-analysis. Gastroenterology.

[38] Newcomb PA, Baron J, Cotterchio M, Gallinger S, Grove J, Haile R (2007). Colon Cancer Family Registry: an international resource for studies of the genetic epidemiology of colon cancer. Cancer Epidemiol.

[39] Wang H, Burnett T, Kono S, Haiman CA, Iwasaki M, Wilkens LR (2014). Trans-ethnic genome-wide association study of colorectal cancer identifies a new susceptibility locus in VTI1A. Nat Commun.

[40] Schmit SL, Edlund CK, Schumacher FR, Gong J, Harrison TA, Huyghe JR, et al. Novel common genetic susceptibility loci for colorectal cancer. J Natl Cancer Inst. 2019;111(2):146-57. 10.1093/jnci/djy099 PMC6555904 29917119

[41] Huyghe JR, Bien SA, Harrison TA, Kang HM, Chen S, Schmit SL, et al. Discovery of common and rare genetic risk variants for colorectal cancer. Nat Genet. 2019;51(1):76-87. 10.1038/s41588-018-0286-6 PMC6358437 30510241

[42] Hernán MA, Robins JM (2020). Causal inference: what if.

[43] Nicola O (2011). A procedure to tabulate and plot results after flexible modeling of a quantitative covariate. Stata J.

[44] Smith-Warner SA, Spiegelman D, Ritz J, Albanes D, Beeson WL, Bernstein L (2006). Methods for pooling results of epidemiologic studies: the Pooling Project of Prospective Studies of Diet and Cancer. Am J Epidemiol.

[45] Sugiyama M, Kanno T, Ohkubo A, Muto Y, Murata K, Ueno Y (1992). The clinical usefulness of the molar ratio of branched-chain amino acids to tyrosine (BTR) in discriminating stage of chronic liver diseases. Rinsho byori Japan J Clin Pathol.

[46] Ishikawa T (2012). Branched-chain amino acids to tyrosine ratio value as a potential prognostic factor for hepatocellular carcinoma. World J Gastroenterol.

[47] Verbanck M, Chen CY, Neale B, Do R (2018). Detection of widespread horizontal pleiotropy in causal relationships inferred from Mendelian randomization between complex traits and diseases. Nat Genet.

[48] Burgess S, Dudbridge F, Thompson SG (2016). Combining information on multiple instrumental variables in Mendelian randomization: comparison of allele score and summarized data methods. Stat Med.

[49] Bowden J, Del Greco MF, Minelli C, Davey Smith G, Sheehan N, Thompson J (2017). A framework for the investigation of pleiotropy in two-sample summary data Mendelian randomization. Stat Med.

[50] Bowden J, Davey Smith G, Burgess S (2015). Mendelian randomization with invalid instruments: effect estimation and bias detection through Egger regression. Int J Epidemiol.

[51] Bowden J, Davey Smith G, Haycock PC, Burgess S (2016). Consistent estimation in Mendelian randomization with some invalid instruments using a weighted median estimator. Genet Epidemiol.

[52] Hartwig FP, Davey Smith G, Bowden J (2017). Robust inference in summary data Mendelian randomization via the zero modal pleiotropy assumption. Int J Epidemiol.

[53] Buckley DB, Klaassen CD (2009). Mechanism of gender-divergent UDP-glucuronosyltransferase mRNA expression in mouse liver and kidney. Drug Metab Dispos.

[54] Stocker R, Yamamoto Y, McDonagh AF, Glazer AN, Ames BN (1987). Bilirubin is an antioxidant of possible physiological importance. Science (New York).

[55] Stocker R (2004). Antioxidant activities of bile pigments. Antioxid Redox Signal.

[56] Rao P, Suzuki R, Mizobuchi S, Yamaguchi T, Sasaguri S (2006). Bilirubin exhibits a novel anti-cancer effect on human adenocarcinoma. Biochem Biophys Res Commun.

[57] Seo MY, Lee SM (2002). Protective effect of low dose of ascorbic acid on hepatobiliary function in hepatic ischemia/reperfusion in rats. J Hepatol.

[58] Bosma PJ (2003). Inherited disorders of bilirubin metabolism. J Hepatol.

[59] Zheng J, Baird D, Borges MC, Bowden J, Hemani G, Haycock P (2017). Recent developments in Mendelian randomization studies. Curr Epidemiol Rep.

[60] van der Logt EM, Bergevoet SM, Roelofs HM, van Hooijdonk Z, te Morsche RH, Wobbes T (2004). Genetic polymorphisms in UDP-glucuronosyltransferases and glutathione S-transferases and colorectal cancer risk. Carcinogenesis..

[61] Wallner M, Marculescu R, Doberer D, Wolzt M, Wagner O, Vitek L (2013). Protection from age-related increase in lipid biomarkers and inflammation contributes to cardiovascular protection in Gilbert’s syndrome. Clin Sci (London).

[62] Seyed Khoei N, Grindel A, Wallner M, Molzer C, Doberer D, Marculescu R (2018). Mild hyperbilirubinaemia as an endogenous mitigator of overweight and obesity: implications for improved metabolic health. Atherosclerosis..

[63] Hernan MA (2010). The hazards of hazard ratios. Epidemiology (Cambridge).

[64] Gkatzionis A, Burgess S (2019). Contextualizing selection bias in Mendelian randomization: how bad is it likely to be?. Int J Epidemiol.

[65] Magno R, Maia AT (2020). gwasrapidd: an R package to query, download and wrangle GWAS catalog data. Bioinformatics (Oxford).

[66] Hemani G, Bowden J, Davey SG (2018). Evaluating the potential role of pleiotropy in Mendelian randomization studies. Hum Mol Genet.

