# Supplementary material for: Circulating bilirubin levels and risk of colorectal cancer: serological and Mendelian randomization analyses
Source: BMC Med. 2020 Sep 3;18:229. doi: 10.1186/s12916-020-01703-w (PMC7469292; doi:10.1186/s12916-020-01703-w)
Supplement: Supplementary file 1 — Additional file 1 Supplementary tables 1–6, supplementary figures 1–3, supplementary text 1. Suppl. table 1 - Summary statistics for the genetic association with bilirubin levels, and sex-stratified colorectal cancer risk. Suppl. table 2 - Participating studies in the genetic consortia (GECCO/ CCFR/ and CORECT). Suppl. table 3 - Associations between unconjugated bilirubin (UCB) levels and colorectal cancer risk after different sensitivity analyses in the EPIC study. Suppl. table 4 - Results for the Mendelian randomization sensitivity analyses. Suppl. table 5 - Results for the Mendelian randomization sensitivity analyses: positive control outcomes for pancreatic cancer. Suppl. table 6 - Results for genome-wide associations of instruments with other phenotypes. Suppl. figure 1 - Cubic spline modeling of unconjugated bilirubin (UCB) levels in relation to colorectal cancer risk in the EPIC study. Suppl. figure 2 - Association between UGT1A1 polymorphism (rs6431625) and unconjugated bilirubin (UCB) levels in the EPIC study (with available GWAS data). Suppl. figure 3 - Directed acyclic graph (DAG) of the causal structure of associations between unconjugated bilirubin (UCB) levels in relation to colorectal cancer risk in the EPIC study. Suppl. text 1 - Specific funding sources and acknowledgements of participating studies. [file 12916_2020_1703_MOESM1_ESM.pdf]

Circulating Bilirubin Levels and Risk of Colorectal Cancer: Serological and Mendelian Randomization Analyses

Nazlisadat Seyed Khoei, Mazda Jenab, Neil Murphy, Barbara L. Banbury, Robert Carreras-Torres, Vivian Viallon, et al.

Corresponding Author:
Heinz Freisling, PhD
International Agency for Research on Cancer (IARC-WHO)
150 cours Albert Thomas
69372 Lyon CEDEX 08, France
Off. +33 472 738 664
E-mail: freislingh@iarc.fr

Additional file 1: Supplementary Tables 1-6, Supplementary Figures 1-3, and Supplementary Text 1

Supplementary Table 1 | Summary statistics for the genetic association with bilirubin levels, and sex-stratified colorectal cancer risk.

Supplementary Table 2 | Participating studies in genetic consortia (GECCO/ CCFR/ and CORECT)

Supplementary Table 3 | The association between unconjugated bilirubin (UCB) levels and colorectal cancer risk after different sensitivity analyses in the EPIC study

Supplementary Table 4 | Results for Mendelian randomization sensitivity analyses

Supplementary Table 5 | Results for the Mendelian randomization sensitivity analyses: positive control outcomes for pancreatic cancer

Supplementary Table 6 | Results for genome-wide associations of instruments with other phenotypes

Supplementary Figure 1 | Cubic spline modelling of unconjugated bilirubin (UCB) levels in relation to colorectal cancer risk in the EPIC study

Supplementary Figure 2 | Association between *UGT1A1* polymorphism (rs6431625) and unconjugated bilirubin (UCB) levels in the EPIC study (with available GWAS data)

Supplementary Figure 3 | Directed acyclic graph (DAG) of the causal structure of associations between unconjugated bilirubin (UCB) levels in relation to colorectal cancer risk in the EPIC study

Supplementary Text 1 | Specific funding sources and acknowledgements of participating studies.

Supplementary Table 1 | Summary statistics for the genetic association with bilirubin levels, and sex-stratified colorectal cancer risk

					Total bilirubin			CRC in men		CRC in women	
**SNP**	**Chr**	**Base pair position**	**Effect allele**	**Reference allele**	**Beta**	**SE**	**Explained variance (%)**	**Beta**	**SE**	**Beta**	**SE**
rs2375279	1	25541931	C	T	0.022	0.004	1.29E-04	-0.009	0.019	0.003	0.019
rs17513135	1	40035686	C	T	0.018	0.003	1.17E-04	-0.028	0.016	0.003	0.016
rs6682423	1	63171063	T	C	0.021	0.003	1.98E-04	0.011	0.015	0.011	0.015
rs1762486	1	107627697	A	G	0.020	0.003	1.78E-04	-0.036	0.015	-0.013	0.015
rs61812598	1	154420087	A	G	0.016	0.003	1.24E-04	-0.003	0.014	-0.025	0.014
rs857725	1	158607935	T	G	0.033	0.003	4.33E-04	0.018	0.015	0.001	0.015
rs788644	1	202256962	G	T	0.018	0.003	1.56E-04	0.001	0.014	0.007	0.014
rs1874121	1	220969049	T	C	0.023	0.003	2.47E-04	-0.024	0.015	-0.006	0.015
rs556107	1	234853059	T	C	0.021	0.003	2.28E-04	0.029	0.014	0.016	0.014
rs13030095	2	26026598	A	G	0.015	0.003	9.79E-05	-0.012	0.015	0.025	0.015
rs2053799	2	32883197	G	A	0.015	0.003	1.03E-04	-0.013	0.014	0.000	0.014
rs4671605	2	64887382	T	C	0.021	0.003	1.65E-04	0.004	0.016	-0.017	0.016
rs6734238	2	113841030	A	G	0.023	0.003	2.46E-04	-0.034	0.014	-0.035	0.014
rs1047891	2	211540507	A	C	0.023	0.003	2.36E-04	0.008	0.015	0.004	0.015
rs6731997	2	232560411	G	A	0.017	0.003	9.89E-05	0.020	0.017	0.028	0.017
rs4973588	2	233834975	A	G	0.035	0.004	2.97E-04	0.002	0.019	0.018	0.019
**rs6431625**	**2**	**234637912**	**C**	**T**	**0.598**	**0.002**	**0.169**	**0.038**	**0.014**	**0.005**	**0.014**
rs10929023	2	235169902	G	A	0.023	0.003	2.14E-04	0.011	0.015	-0.002	0.015
rs2267846	3	48556339	A	G	0.017	0.003	1.08E-04	0.005	0.018	0.016	0.018
rs9826148	3	114464858	C	T	0.026	0.004	1.28E-04	0.046	0.021	-0.006	0.021
rs6779903	3	135720851	T	G	0.019	0.003	1.44E-04	-0.011	0.015	-0.029	0.015
rs1052618	3	136574501	A	G	0.028	0.003	3.45E-04	-0.012	0.015	-0.010	0.015

rs1482852	3	156798294	A	G	0.017	0.003	1.32E-04	0.006	0.014	0.001	0.014
rs61791066	3	170713380	C	T	0.031	0.004	2.06E-04	-0.031	0.020	-0.030	0.020
rs11917973	3	195838613	T	C	0.014	0.003	9.48E-05	-0.025	0.015	0.018	0.014
rs13092376	3	196516288	C	A	0.014	0.003	9.51E-05	0.022	0.014	0.006	0.014
rs115558925	4	69765782	A	C	0.029	0.004	1.55E-04	-0.039	0.025	0.028	0.025
rs151450	4	88016514	G	A	0.017	0.003	1.45E-04	-0.009	0.014	-0.024	0.014
rs1126673	4	100045616	C	T	0.020	0.003	1.66E-04	0.015	0.015	-0.019	0.015
rs79800919	4	124787383	G	A	0.026	0.004	1.36E-04	0.030	0.021	0.006	0.021
rs11727331	4	145091680	A	G	0.040	0.006	1.55E-04	0.032	0.032	0.035	0.032
rs10003923	4	175086079	C	T	0.015	0.003	1.14E-04	0.006	0.014	-0.007	0.014
rs6869704	5	122833667	T	C	0.017	0.003	1.37E-04	-0.001	0.014	-0.004	0.014
rs274555	5	131722951	C	T	0.018	0.003	1.51E-04	-0.001	0.014	0.021	0.014
rs12515233	5	158417109	C	A	0.019	0.003	1.05E-04	-0.007	0.018	-0.025	0.018
rs72835688	6	12811955	C	T	0.030	0.005	1.02E-04	0.005	0.032	0.001	0.032
rs9379764	6	25414023	G	T	0.018	0.003	1.23E-04	-0.003	0.016	0.012	0.016
rs198851	6	26104632	T	G	0.047	0.003	5.59E-04	-0.011	0.019	0.010	0.019
rs200484	6	27775674	A	G	0.023	0.004	1.23E-04	-0.030	0.023	-0.064	0.023
rs853684	6	28294550	T	C	0.024	0.003	2.84E-04	-0.006	0.014	-0.003	0.014
rs9267488	6	31514247	A	G	0.022	0.004	1.22E-04	-0.053	0.023	-0.039	0.022
rs2395943	6	42940673	A	G	0.020	0.003	1.93E-04	-0.006	0.014	-0.004	0.014
rs12210538	6	110760008	G	A	0.017	0.003	9.99E-05	0.003	0.016	-0.004	0.016
rs1490384	6	126851160	C	T	0.019	0.003	1.79E-04	0.024	0.014	0.016	0.014
rs11753995	6	160575366	G	A	0.025	0.003	1.71E-04	-0.006	0.019	0.026	0.019
rs4410790	7	17284577	T	C	0.031	0.003	4.43E-04	-0.022	0.014	-0.017	0.014
rs55696093	7	21605973	G	A	0.026	0.003	2.31E-04	-0.018	0.017	0.022	0.017
rs57720904	7	28180099	C	T	0.030	0.005	1.36E-04	-0.007	0.021	0.005	0.022
rs58699591	7	97876704	C	T	0.023	0.003	1.39E-04	0.008	0.019	0.002	0.019
rs33963055	8	23407178	T	C	0.019	0.003	1.07E-04	0.002	0.018	0.002	0.018
rs34664882	8	41543675	A	G	0.076	0.007	3.85E-04	0.018	0.044	0.066	0.044

rs2923430	8	42391195	C	T	0.018	0.003	1.57E-04	-0.010	0.014	-0.011	0.014
rs8192870	8	59412066	T	G	0.015	0.003	9.94E-05	-0.040	0.014	0.003	0.014
rs6986601	8	131026260	G	A	0.014	0.003	9.66E-05	-0.026	0.014	-0.013	0.014
rs79170239	9	94044272	T	C	0.035	0.003	3.78E-04	-0.019	0.017	-0.036	0.017
rs3118753	9	97051962	C	T	0.014	0.003	9.68E-05	-0.028	0.014	0.011	0.014
rs2519093	9	136141870	C	T	0.024	0.003	1.76E-04	-0.024	0.016	-0.007	0.017
rs34755157	9	137113606	C	T	0.023	0.003	1.52E-04	0.049	0.018	0.004	0.018
rs10761756	10	65172328	C	T	0.040	0.003	7.81E-04	0.001	0.014	-0.014	0.014
rs17476364	10	71094504	C	T	0.091	0.004	1.64E-03	-0.026	0.024	-0.006	0.024
rs2901610	10	94536864	C	T	0.022	0.003	2.47E-04	-0.005	0.014	0.031	0.014
rs12768009	10	96525865	G	A	0.024	0.004	1.46E-04	-0.012	0.019	-0.015	0.019
rs2792751	10	113940329	T	C	0.021	0.003	1.81E-04	-0.009	0.015	-0.025	0.015
rs2245095	10	122857596	C	T	0.048	0.004	3.65E-04	-0.019	0.023	-0.010	0.023
rs11601507	11	5701074	A	C	0.028	0.005	1.03E-04	-0.027	0.026	0.017	0.026
rs10832027	11	13357183	G	A	0.024	0.003	2.48E-04	0.004	0.014	0.010	0.014
rs174554	11	61579463	A	G	0.021	0.003	2.02E-04	0.068	0.015	0.067	0.015
rs499974	11	75455021	A	C	0.029	0.003	2.18E-04	-0.001	0.018	0.007	0.018
rs717662	11	100493995	T	C	0.026	0.004	1.34E-04	0.034	0.021	0.000	0.021
rs3741298	11	116657561	C	T	0.032	0.003	3.11E-04	0.023	0.017	0.009	0.017
rs76895963	12	4384844	G	T	0.062	0.010	1.54E-04	0.232	0.056	0.008	0.056
rs73080739	12	20761863	G	A	0.033	0.004	1.91E-04	-0.049	0.022	-0.028	0.022
rs4149056	12	21331549	C	T	0.183	0.003	8.57E-03	-0.007	0.018	-0.001	0.018
rs1283809	12	21980618	C	T	0.021	0.003	1.16E-04	0.013	0.018	-0.006	0.018
rs36124182	12	24214934	A	G	0.049	0.007	1.68E-04	-0.002	0.033	-0.007	0.033
rs4760682	12	48512285	C	A	0.017	0.003	9.22E-05	-0.044	0.019	-0.007	0.019
rs10876376	12	53261822	A	G	0.016	0.003	1.31E-04	-0.011	0.014	-0.004	0.014
rs2657879	12	56865338	G	A	0.018	0.003	9.46E-05	-0.001	0.017	-0.013	0.018
rs3184504	12	111884608	T	C	0.038	0.003	7.25E-04	-0.071	0.014	-0.070	0.014
rs12811045	12	115527503	A	G	0.017	0.003	9.89E-05	0.008	0.017	0.017	0.017

rs7135337	12	121404155	C	A	0.016	0.003	1.20E-04	-0.009	0.014	-0.020	0.014
rs139763750	14	51049397	A	G	0.038	0.006	1.45E-04	-0.020	0.032	-0.044	0.032
rs61984409	14	64730021	A	C	0.015	0.003	9.73E-05	0.020	0.014	0.026	0.014
rs339969	15	60883281	C	A	0.026	0.003	3.20E-04	-0.051	0.014	0.009	0.014
rs17184256	15	63785421	G	A	0.022	0.003	2.14E-04	0.037	0.015	0.014	0.015
rs67257650	15	73979507	T	C	0.017	0.003	1.42E-04	-0.028	0.014	-0.029	0.014
rs181207	16	28513530	C	T	0.020	0.003	1.76E-04	0.000	0.015	-0.021	0.015
rs4575545	16	79755446	G	A	0.017	0.003	1.22E-04	0.055	0.015	-0.013	0.015
rs7185774	16	81249927	C	T	0.015	0.003	9.57E-05	0.012	0.014	-0.004	0.014
rs247827	16	84582825	G	A	0.019	0.003	1.10E-04	-0.022	0.018	-0.002	0.018
rs2968478	16	88858646	T	G	0.023	0.003	2.60E-04	-0.008	0.014	0.005	0.014
rs7222046	17	7806529	G	A	0.017	0.003	1.34E-04	-0.032	0.014	-0.017	0.014
rs1045599	17	15879910	C	T	0.016	0.003	1.24E-04	0.017	0.014	-0.010	0.014
rs7213285	17	27206029	A	G	0.020	0.003	1.07E-04	0.000	0.019	-0.003	0.019
rs1477141	17	53361838	G	T	0.014	0.003	1.03E-04	-0.007	0.014	0.015	0.014
rs764424	17	57505604	G	A	0.015	0.003	1.12E-04	0.005	0.014	0.005	0.014
rs11079420	17	59253051	A	G	0.020	0.003	1.33E-04	0.032	0.016	0.043	0.016
rs7223257	17	61787684	T	C	0.017	0.003	1.33E-04	-0.013	0.015	-0.001	0.015
rs12601919	17	65825374	A	G	0.021	0.003	1.35E-04	-0.001	0.017	-0.017	0.017
rs2909210	17	66456481	T	G	0.015	0.003	1.17E-04	0.016	0.014	0.018	0.014
rs1135688	17	73827205	C	T	0.015	0.003	9.06E-05	-0.023	0.015	0.017	0.015
rs379149	17	76126586	C	T	0.027	0.004	1.54E-04	-0.010	0.024	0.013	0.023
rs112896133	19	12429782	G	A	0.078	0.011	1.55E-04	-0.038	0.070	0.144	0.069
rs12609744	19	12994140	T	C	0.016	0.003	1.10E-04	0.008	0.016	-0.013	0.016
rs59616136	19	17252041	A	G	0.036	0.004	2.15E-04	0.014	0.023	0.013	0.023
rs58542926	19	19379549	T	C	0.041	0.005	2.34E-04	-0.018	0.026	0.021	0.026
rs157595	19	45425460	G	A	0.017	0.003	1.31E-04	-0.008	0.014	0.018	0.014
rs676388	19	49211969	C	T	0.021	0.003	2.29E-04	-0.003	0.014	0.024	0.014
rs12459419	19	51728477	T	C	0.015	0.003	9.91E-05	0.005	0.015	-0.011	0.015

rs117113213	20	39165692	G	A	0.046	0.007	1.32E-04	0.033	0.043	0.029	0.044
rs6129760	20	39746403	G	A	0.018	0.003	1.37E-04	-0.002	0.015	0.007	0.015
rs1800961	20	43042364	T	C	0.062	0.007	2.38E-04	-0.027	0.040	-0.069	0.041
rs4820091	22	21940189	T	G	0.018	0.003	9.78E-05	-0.007	0.017	0.054	0.017
rs855791	22	37462936	G	A	0.028	0.003	3.94E-04	0.022	0.014	0.013	0.014
rs738408	22	44324730	T	C	0.019	0.003	1.19E-04	0.001	0.016	0.010	0.016

Abbreviations: Chr: chromosome, SE: standard error
Position in Genome Reference Consortium Human Build 37 (GRCh37). Effect and standard error of inverse-normal-transformed bilirubin levels.
Total bilirubin can be regarded as the sum of conjugated and unconjugated bilirubin (UCB).
The main SNP (rs6431625) is in strong linkage equilibrium with a homozygous insertion of TA pairs (genotype *UGT1A1*28/*28)* in the *UGT1A1* gene promoter. UGT1A1 glucuronidates bilirubin in the liver and results in the formation of bilirubin mono- and di-glucuronide which are excreted into bile. This polymorphism results in reduced bilirubin glucuronidation activity and leads to hereditary unconjugated hyperbilirubinemia.

Supplementary Table 2 | Participating studies in the genetic consortia (GECCO/ CCFR/ and CORECT)

Study	n total	n cases	n controls	n men (cases/controls)	n women (cases/controls)
ASTERISK	1839	892	947	552/524	340/423
ATBC	177	147	30	147/30	0/0
CCFR_1	2014	1036	978	536/469	500/509
CCFR_2	716	331	385	152/176	179/209
CCFR_3	1851	1190	661	582/303	608/358
CCFR_4	2124	1590	534	956/287	634/247
CLUEII	518	258	260	121/119	137/141
COLO2&3	211	87	124	47/70	40/54
COLOCARE_HEIDELBERG	223	187	36	124/17	63/19
COLOCARE_SEATTLE	169	169	0	96/0	73/0
COLON	1335	643	692	408/436	235/256
CORSA_1	2234	1460	774	915/442	545/332
CORSA_2	2483	1210	1273	784/822	426/451
CPSII_1	1076	540	536	272/276	268/260
CPSII_2	688	339	349	163/165	176/184
CRCGEN	1546	760	786	491/420	269/366
CZECH	3293	1675	1618	1051/883	624/735
DACHS_1	3409	1707	1702	999/1017	708/685
DACHS_2	1164	666	498	406/323	260/175
DACHS_3	1827	1210	617	754/375	456/242
DALS_1	1411	702	709	399/400	303/309
DALS_2	863	402	461	212/241	190/220
EDRN	589	273	316	166/155	107/161
EPIC	4401	2095	2306	979/1074	1116/1232
EPICOLON	609	267	342	157/201	110/141
ESTHER_VERDI	817	397	420	261/273	136/147

HAWAIICCS_AD	628	85	543	49/352	36/191
HCES-CRC	5294	3026	2268	1960/909	1066/1359
HPFS_1	455	227	228	227/228	0/0
HPFS_2	348	176	172	176/172	0/0
HPFS_3_AD	655	312	343	312/343	0/0
HPFS_4	380	183	197	183/197	0/0
HPFS_5_AD	260	155	105	155/105	0/0
KENTUCKY	2167	1035	1132	511/559	524/573
LCCS	2183	1482	701	884/330	598/371
MCCS	1343	709	634	366/325	343/309
MEC_1	672	326	346	178/183	148/163
MEC_2	144	63	81	28/43	35/38
MECC_1	978	483	495	265/262	218/233
MECC_2	1901	1093	808	544/406	549/402
MECC_3	4380	2570	1810	1395/970	1175/840
MSKCC	68	68	0	27/0	41/0
NCCCSI	720	251	469	140/264	111/205
NCCCSII	1281	595	686	354/427	241/259
NFCCR	660	193	467	120/270	73/197
NGCCS	1103	1103	0	621/0	482/0
NHS_1	1165	391	774	0/0	391/774
NHS_2	339	158	181	0/0	158/181
NHS_3_AD	1090	513	577	0/0	513/577
NHS_4	611	308	303	0/0	308/303
NHS_5_AD	477	251	226	0/0	251/226
NHSII	167	87	80	0/0	87/80
NSHDS	829	416	413	149/148	267/265
OFCCR	1116	594	522	242/295	352/227
OSUMC	5527	3094	2433	1666/961	1428/1472

PHS	764	375	389	375/389	0/0
PLCO_1	2496	524	1972	296/1536	228/436
PLCO_2	889	475	414	271/239	204/175
PLCO_4_AD	2105	797	1308	460/571	337/737
PMH-CCFR	398	276	122	0/0	276/122
SEARCH	4288	4173	115	2384/69	1789/46
SELECT	533	264	269	264/269	0/0
SLRCCS	4785	2504	2281	1369/1196	1135/1085
SMC_COSM	1397	566	831	331/483	235/348
SMS_AD	171	41	130	25/57	16/73
USC_HRT_CRC	708	321	387	0/0	321/387
VITAL	565	279	286	150/148	129/138
WHI_1	1991	468	1523	0/0	468/1523
WHI_2	1984	978	1006	0/0	978/1006
WHI_3	1113	554	559	0/0	554/559
TOTAL	**98,715**	**52,755**	**45,940**	**28,270/22,204**	**24,568/23,736**

Abbreviations: ASTERISK: Association Study Evaluating RISK for Sporadic Colorectal Cancer; CCFR: Colon Cancer Family Registry; CCFR: Colon Cancer Family Registry; Colo 2&3: a case-control study from the University of Hawai'I; DACHS: Darmkrebs: Chancen der Verhutüng durch Screening Study; DALS: Diet, Activity and Lifestyle Study; GECCO: Genetics and Epidemiology of Colorectal Cancer Consortium; HPFS: Health Professionals Follow-up Study; MEC: Multiethnic Cohort; NHS: Nurses' Health Study; OFCCR: Ontario Familial Colorectal Cancer Registry; PHS: Physicians' Health Study; PLCO: Prostate, Lung, Colorectal and Ovarian Cancer Screening Trial; PMH: Postmenopausal Hormone Study; VITAL: Vitamins and Lifestyle Study; Women's Health Initiative; CPSII: Cancer Society Cancer Prevention Study II; MECC: Molecular Epidemiology of Colorectal Cancer Study; MCCS: Melbourne Case-Control Study (in Melbourne Collaborative Cohort); NFCCR: NewFoundland Case-Control Study; SEARCH: Studies of Epidemiology and Risk Factors in Cancer Heredity; SWEDEN_Wolk: The Cohort of Swedish Men and the Swedish Mammography CohortThe Swedish Mammography Cohort; ATBC: Alpha-Tocopherol, Beta-Carotene Cancer Prevention; ColoCare_heidelberg: The ColoCare study at the University Hospital Heidelberg; ColoCare_Seattle: The ColoCare study at the Fred Hutchinson Cancer Research Center; ESTHER_VERDI: ESTHER/VERDI study in Saarland, Germany; Kiel: Sample collected in PopGen Biobank; MSKCC: Memorial Sloan Kettering (MSK) cohort; NHS2: Nurses' Health Study II; SWEDEN_Lindblom: The Swedish Low-Risk Colorectal Cancer Study; USC_HRT_CRC: a case-control study for HRT use and CRC risk in Los Angeles county. EPIC: European Prospective Investigation into Cancer and Nutrition

Supplementary Table 3 | The association between unconjugated bilirubin (UCB) levels and colorectal cancer risk after different sensitivity analyses in the EPIC study

Colorectal cancer						
Confounders/ mediators	**Men**			**Women**		
	**n cases/ controls**	**odds ratio (95% CI)**	**P**	**n cases/ controls**	**odds ratio (95% CI)**	**P**
Adjusted Model	**658/658**	**1.19 (1.04-1.36)**	**0.01**	**728/728**	**0.86 (0.76-0.97)**	**0.02**
Model S1	452/452	1.23 (1.04-1.45)	0.02	442/442	0.95 (0.81-1.11)	0.5
Model S2	517/517	1.26 (1.08-1.47)	0.003	570/570	0.87 (0.75-1.00)	0.05
Model S3	297/297	1.26 (1.03-1.55)	0.02	264/264	0.83 (0.68-1.02)	0.08
Model S4	655/655	1.20 (1.05-1.37)	0.01	382/382	0.91 (0.76-1.08)	0.3
Model S5	655/655	1.20 (1.05-1.37)	0.01	727/727	0.87 (0.77-0.98)	0.02
Model S6	423/423	1.11 (0.94-1.31)	0.2	492/492	0.82 (0.70-0.94)	0.01
Model S7	619/619	1.21 (1.05-1.38)	0.01	667/667	0.87 (0.77-0.99)	0.03

Abbreviations: n= number, CI: Confidence Interval, P: P-value

EPIC (European Prospective Investigation into Cancer and Nutrition): Conditional logistic regression models were used to estimate odds ratios (OR) and 95% confidence intervals (CI) for association between log-transformed UCB levels (log-UCB), standardized per one standard deviation (1-SD) increments, and CRC risk. Crude model which was conditioned on the matching factors including, study center, age at blood collection (1 year), fasting status and time of blood collection (3 h) at blood collection, among women, additionally by menopausal status (pre-, peri-, post-menopausal or surgically menopausal), and hormone therapy (HT) (yes, no) at time of blood collection. Multivariable model adjusted for level of education (none/primary school, technical/professional, secondary school, university degree), BMI, height, smoking status (never, former, current smoker), physical activity (inactive, moderately inactive, moderately active, active), alcohol consumption (g/d), dietary intakes of fiber (g/d), red meat (g/d), processed meats (g/d), dairy products (g/d), total energy intake (kcal/d).

Spearman partial correlation coefficients (adjustment for age, fasting status and study center) were calculated in control participants to assess the correlations between baseline log-UCB concentrations, lipid and sugar metabolism (TG, HDL, LDL, Chol, apo A, apo B, HbA1c, C-peptide), oxidative stress biomarkers (reactive oxygen metabolites, ROM and FRAP), inflammatory indicator (hsCRP, adiponectin, leptin), insulin-like growth factor 1 (IGF1) and IGF binding proteins (IGF-BP) (3), dietary factors. Variables with P< 0.1 considered as potential confounders/mediators in this tables.

Model S1 Excluding participants with chronic disease (diabetes and CVD) at baseline

Model S2 Excluding participants with lower than 2 years of follow-up

Model S3 Adjusting for lipid metabolism (TG) and sugar metabolism (HbA1c and c-peptide),

Model S4 Adjusting for oxidative stress (ROM), IGF1and IGF-BP, for inflammatory indicator (leptin and CRP)

Model S5 Adjusting for dietary factors: fruit, vegetable, rice/pasta/other grains, and coffee/tea/herbal tea

Model S6 Adjusting for endotoxins (lipopolysaccharide IgG and lipopolysaccharide IgGA)

Model S7 Excluding missing values in main covariates

The impact of liver function on bilirubin levels is important to assess. We had access to the Biocrates data on these subjects, therefore, we could envision adjusting for liver function using the Fischer Ratio (the molar concentration ratio of BCAAs/AAAs: branched chain amino acids (BCAAs): leucine, valine, isoleucine/aromatic amino acids (AAAs): phenylalanine, tyrosine) and BTR (Branched-chain amino-acids to Tyrosine Ratio). The normal range of Fischer's ratio is between 3.0 and 4.2 and normal range of BTR is between 5.71-9.42 in healthy individuals. Both indexes decrease with increasing severity of hepatic damage and are important for assessing liver metabolism, hepatic functional reserve and the severity of liver dysfunction. They could help for excluding those subjects having liver disease, therefore, we could be sure that participants who had higher UCB suffered not from liver problems. After considering both indexes in our data, nobody had known liver disease.

Supplementary Table 4 | Results for the Mendelian randomization sensitivity analyses

			Likelihood-based method			Inverse-variance weighted method*		Weigthed median approach		Modal-based approach		MR-Egger Simex approach		MR-Egger intercept approach				MR-PRESSO approach		
Colorectal cancer	SNP set	n SNP	odds ratio (95% CI)	P	P_{het}	odds ratio (95% CI)	P	odds ratio (95% CI)	P	odds ratio (95% CI)	P	odds ratio (95% CI)	P	Est	LCI	UCI	P	P_{Global}	$P_{Distortion}$	Outlier SNPs
Men	All	115	1.04 (1.00-1.08)	0.08	3.6E-05	1.04 (0.98-1.10)	0.17	1.06 (1.02-1.11)	0.01	1.05 (1.00-1.10)	0.03	1.07 (1.01-1.13)	0.03	-0.005	-0.008	-0.001	0.01	<1e-04	-	-
	rs6431625	1	1.07 (1.02-1.12)	6.3E-03																
	rs6431625 out	114	0.89 (0.80-1.00)	0.05	1.5E-04	0.90 (0.78-1.03)	0.11	0.96 (0.79-1.16)	0.67	0.92 (0.77-1.10)	0.35	0.95 (0.74-1.22)	0.70	-0.002	-0.007	0.003	0.45	3.0E-04	-	-
	Pleiotropy out	91	0.84 (0.72-0.98)	0.02	3.2E-04	0.84 (0.70-1.02)	0.08	0.78 (0.61-0.98)	0.03	0.79 (0.55-1.12)	0.18	0.91 (0.52-1.60)	0.74	-0.002	-0.011	0.007	0.65	2.0E-04	-	-
Women	All	115	1.01 (0.97-1.05)	0.75	0.03	1.01 (0.96-1.06)	0.78	1.01 (0.96-1.06)	0.75	1.01 (0.96-1.06)	0.75	1.01 (0.96-1.06)	0.79	-1.4E-05	-0.003	0.003	0.99	0.10	-	-
	rs6431625	1	1.01 (0.96-1.06)	0.72																
	rs6431625 out	114	1.00 (0.89-1.11)	0.96	0.03	1.00 (0.88-1.13)	0.96	0.99 (0.83-1.20)	0.96	1.01 (0.84-1.20)	0.93	0.98 (0.78-1.22)	0.83	0.001	-0.005	0.006	0.83	0.04	-	-
	Pleiotropy out	91	1.01 (0.87-1.17)	0.95	0.03	1.00 (0.85-1.19)	0.96	0.94 (0.73-1.21)	0.66	0.95 (0.65-1.37)	0.77	0.90 (0.54-1.50)	0.69	0.002	-0.007	0.011	0.61	0.03	-	-

Abbreviations: SNP: single nucleotide polymorphism, CI: Confidence Interval, P: P-value, P_{het}: P-value for SNP heterogeneity,
*Multiplicative random effects.

Supplementary Table 5 | Results for the Mendelian randomization sensitivity analyses: positive control outcomes for pancreatic cancer

			Likelihood-based method			Weigthed median approach		MR-Egger Simex approach	
**Pancreatic cancer**	**SNP set**	**n SNP**	**odds ratio (95% CI)**	**P**	**P_{het}**	**odds ratio (95% CI)**	**P**	**odds ratio (95% CI)**	**P**
**Men**	rs6431625	1	1.06 (0.95-1.18)	0.27					
	rs6431625 out	113	0.75 (0.58-0.96)	0.02	2.50E^{-03}	0.60 (0.39-0.93)	0.02	0.60 (0.35-1.05)	0.07
	Pleiotropy out	91	0.89 (0.63-1.27)	0.53	0.11	0.96 (0.56-1.64)	0.89	0.89 (0.29-2.73)	0.83
**Women**	rs6431625	1	0.98 (0.87-1.10)	0.71					
	rs6431625 out	113	0.73 (0.55-0.97)	0.03	0.01	0.78 (0.49-1.26)	0.32	0.81 (0.14-1.49)	0.49
	Pleiotropy out	91	0.81 (0.55-1.20)	0.29	0.11	0.96 (0.52-1.76)	0.90	1.44 (0.42-4.97)	0.56

Abbreviations: SNP: single nucleotide polymorphism, CI: Confidence Interval, P: P-value, P_{het}: P-value for SNP heterogeneity
Men: n cases= 3861; n controls= 4056; minimum detectable odds ratio *UGT1A1* SNP=1.17/0.86; non-*UGT1A1* SNPs= 1.43/0.70.
Women: n cases= 3252; n controls= 3268; minimum detectable odds ratio *UGT1A1* SNP= 1.18/0.84; non-*UGT1A1* SNPs= 1.48/0.67.

References: Pancreatic Cancer Cohort Consortium (PanScan; 12 studies) and the Pancreatic Cancer Case-Control Consortium (PanC4; 10 studies) through the National Center for Biotechnology Information database of Genotypes and Phenotypes (dbGaP; Study Accession: phs000206.v3.p2 and phs000648.v1.p1; project reference #9314),
Petersen GM, Amundadottir L, Fuchs CS, Kraft P, Stolzenberg-Solomon RZ, Jacobs KB, et al. A genome-wide association study identifies pancreatic cancer susceptibility loci on chromosomes 13q22.1, 1q32.1 and 5p15.33. Nature genetics. 2010;42(3):224-8;
Amundadottir L, Kraft P, Stolzenberg-Solomon RZ, Fuchs CS, Petersen GM, Arslan AA, et al. Genome-wide association study identifies variants in the ABO locus associated with susceptibility to pancreatic cancer. Nature genetics. 2009;41(9):986-90.
Childs EJ, Mocci E, Campa D, Bracci PM, Gallinger S, Goggins M, et al. Common variation at 2p13.3, 3q29, 7p13 and 17q25.1 associated with susceptibility to pancreatic cancer. Nature genetics. 2015;47(8):911-6.

Supplementary Table 6 | Results for genome-wide associations of instruments with other phenotypes

Trait	P	SNP
2-methylbutyrylglycine	3.00E-22	rs1047891
2-methylbutyrylglycine, 3-methylcrotonylglycine, indoleacetylglutamine, indoleacetylglycine, isobutyrylglycine, isovalerylglutamine, isovalerylglycine, tigloylglycine, X-17688	2.00E-16	rs1047891
3-hydroxy-3-methylglutarate, 3-methylglutaconate, 3-methylglutarate, 2-methylglutarate, 3-methylglutarylcarnitine	8.00E-30	rs1047891
3-methylcrotonylglycine	4.00E-14	rs1047891
3-methylglutaconate	3.00E-25	rs1047891
3-methylglutarate/2-methylglutarate	5.00E-26	rs1047891
3-methylglutarylcarnitine 2	2.00E-21	rs1047891
Education attainment	4.00E-12	rs1047891
Glycine	1.00E-300	rs1047891
Hexanoylglycine	9.00E-23	rs1047891
Hexanoylglycine, isocaproylglycine, X-16567, X-24402	3.00E-24	rs1047891
Isobutyrylglycine	7.00E-18	rs1047891
Isocaproylglycine	1.00E-25	rs1047891
Isovalerylglycine	4.00E-12	rs1047891
Tigloylglycine	7.00E-17	rs1047891
X - 16567	3.00E-13	rs1047891
X - 24402	1.00E-12	rs1047891
BMI-adjusted	3.00E-08	rs10832027
Education attainment	1.00E-15	rs11753995
Acetylcarnitine	8.00E-29	rs12210538
Acylcarnitine total	7.00E-37	rs12210538
Carnitine free	6.00E-25	rs12210538
Leucine|Isoleucine / Propionylcarnitine	2.00E-26	rs12210538
Octadecadienylcarnitine	5.00E-21	rs12210538
Palmitoylcarnitine	7.00E-31	rs12210538
Propionylcarnitine	3.00E-25	rs12210538
Myeloid cell surface antigen CD33	3.00E-165	rs12459419
Education attainment	2.00E-12	rs1482852
BMI-adjusted	5.00E-12	rs1490384
Total brain volume	2.00E-15	rs1490384
Education attainment	6.00E-49	rs174554
Phosphatidylcholine with diacyl residue sum C38:3/Phosphatidylcholine with acyl-alkyl residue sum C40:1	3.00E-17	rs174554
Education attainment	1.00E-16	rs17513135
BMI-adjusted	6.00E-19	rs1800961
Education attainment	5.00E-23	rs1800961
Education attainment	3.00E-33	rs198851
Adhesion G protein-coupled receptor F5, ADGRF5.6409.57.3	1.00E-155	rs2519093
C1GALT1-specific chaperone 1, C1GALT1C1.5735.54.3	2.00E-89	rs2519093

D-glucuronyl C5-epimerase, GLCE.7808.5.3	1.00E-78	rs2519093
Education attainment	2.00E-48	rs2519093
E-selectin, SELE.3470.1.2	0	rs2519093
Interleukin-3 receptor subunit alpha, IL3RA.13744.37.3	8.00E-198	rs2519093
N-acetyllactosaminide beta-1,3-N-acetylglucosaminyltransferase 2, B3GNT2.7980.72.3	4.00E-23	rs2519093
Protein FAM3B, FAM3B.9177.6.3	3.00E-20	rs2519093
P-selectin, SELP.4154.57.2	4.00E-48	rs2519093
Thrombospondin type-1 domain-containing protein 1, THSD1.5621.64.3	3.00E-19	rs2519093
Vascular endothelial growth factor receptor 3, FLT4.2358.19.2	6.00E-80	rs2519093
Glutamine	3.00E-70	rs2657879
Education attainment	1.00E-09	rs3184504
Kynurenine	6.00E-18	rs3184504
Vascular cell adhesion protein 1, VCAM1.2967.8.1	2.00E-14	rs3184504
Education attainment	5.00E-268	rs3741298
Triglyceride	3.00E-08	rs3741298
1-arachidonoylglycerophosphoethanolamine	3.00E-18	rs4149056
4-androsten-3beta,17beta-diol disulfate 2	1.00E-18	rs4149056
C18:1-DC	2.00E-13	rs4149056
C18-DC:C20-OH	2.00E-12	rs4149056
Glycocholenate sulfate	4.00E-32	rs4149056
Isoleucine/X-11529	0	rs4149056
LPE 20:4	1.00E-10	rs4149056
X-21470	2.00E-11	rs4149056
X-11491	3.00E-32	rs4149056
X-11538	1.00E-37	rs4149056
X-12063	1.00E-73	rs4149056
X-12456	8.00E-17	rs4149056
X-13429	5.00E-22	rs4149056
Education attainment	2.00E-30	rs4410790
Education attainment	7.00E-13	rs556107
Education attainment	4.00E-37	rs58542926
Vitamin E alpha tocopherol	1.00E-08	rs58542926
BMI-adjusted	2.00E-126	rs61812598
Interleukin-6 receptor	6.00E-63	rs61812598
BMI-adjusted	6.00E-38	rs6734238
Education attainment	6.00E-19	rs6734238
Education attainment	5.00E-14	rs855791
hemoglobin concentration	2.00E-51	rs855791
Hemoglobin	3.00E-08	rs855791
Iron	9.00E-11	rs855791
Mean corpuscular volume (MCV)	1.00E-10	rs855791
Transferrin receptor protein 1, TFRC.8795.48.3	2.00E-16	rs855791

Supplementary Figure 1 | Cubic spline modelling of unconjugated bilirubin (UCB) levels in relation to colorectal cancer risk in the EPIC study

A. Colorectal cancer risk in men

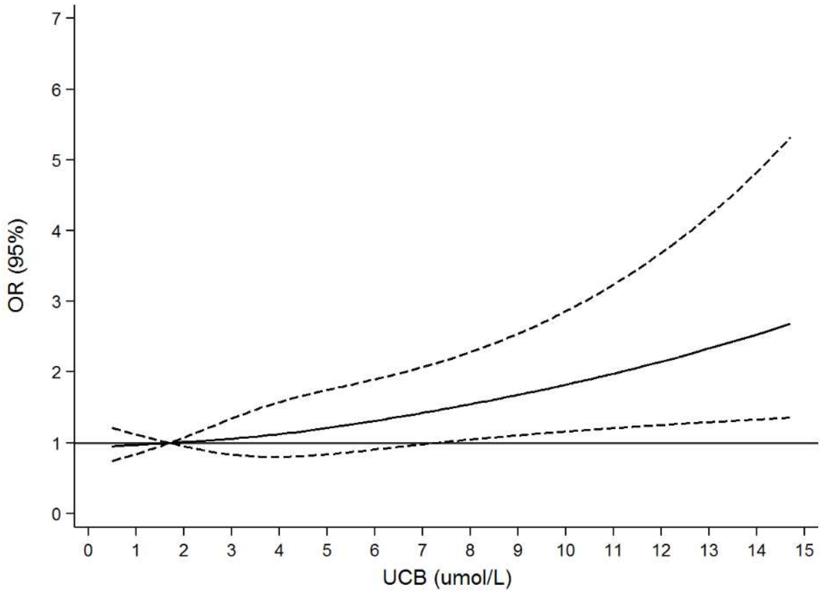

B. Colorectal cancer risk in women

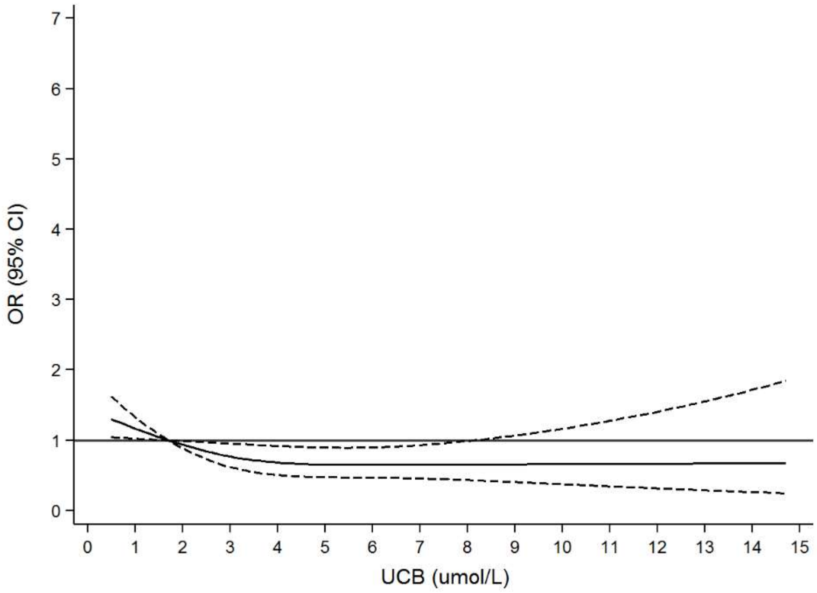

This graph shows multivariable adjusted odds ratio (solid line) with 95% confidence interval (dashed lines) for the association of UCB levels (µmol/L) to the incident CRC in in the EPIC nested case-control study in men (A) and women (B). It was modeled by restricted cubic splines with 3 knots at percentiles 10th, 50th, and 90th in a logistic regression model to evaluate the linearity hypothesis.
$P_{nonlinearity}$ (men)= 0.7, $P_{nonlinearity}$ (women)= 0.1

Supplementary Figure 2 | Association between *UGT1A1* polymorphism (rs6431625) and unconjugated bilirubin (UCB) levels in the EPIC study (with available GWAS data)

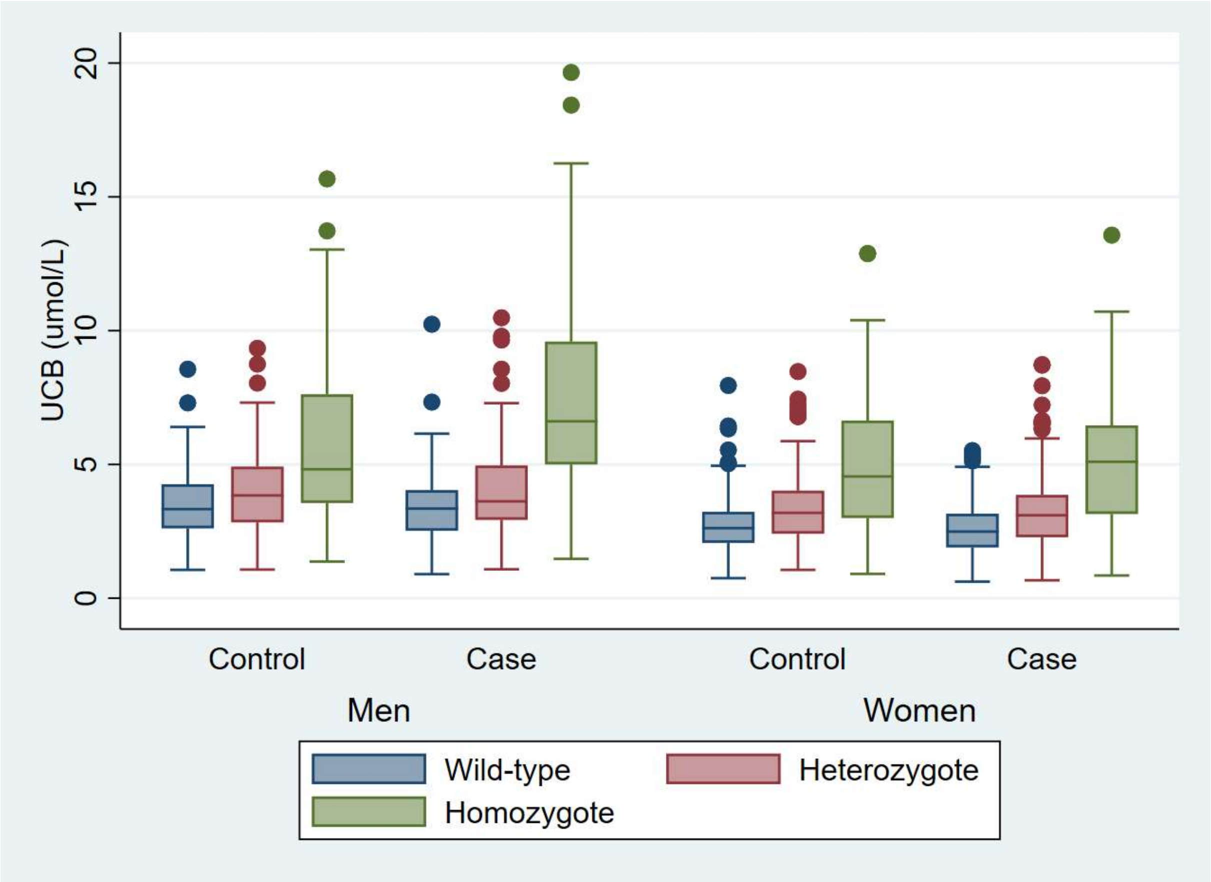

Linear regression (adjusted for sex) were calculated in all controls. We observed significant association between UCB concentrations and rs6431625 (R^2= 0.20, P< 0.001, n controls= 808).
The comparison between groups statistically analyzed using one-way analyses of variance (ANOVA) to see association between variables.

Supplementary Figure 3 | Directed acyclic graph (DAG) of the causal structure of associations between unconjugated bilirubin (UCB) levels in relation to colorectal cancer risk in the EPIC study

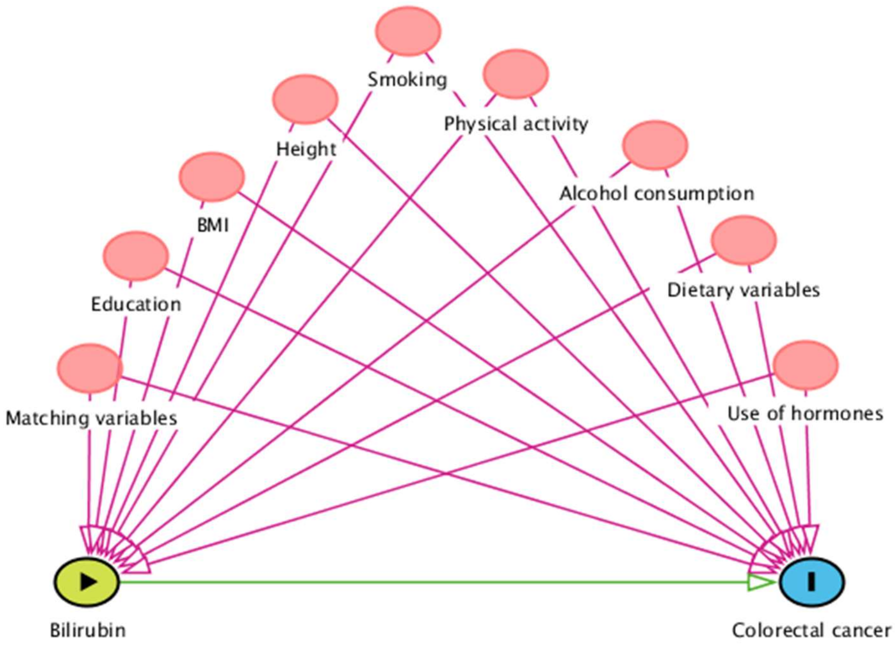

Matching variables: sex, age at blood collection, study center, time of day at blood collection, fasting status, menopausal status, and phase of menstrual cycle at blood collection.
BMI (body mass index) indicating obesity replaced by waist circumference resulted in virtually identical risk associations.
Dietary variables modelled separately: dietary intakes of fiber (g/d), red meat (g/d), processed meats (g/d), dairy products (g/d), and total energy intake (kcal/d).

Reference and software: Johannes Textor, Benito van der Zander, Mark K. Gilthorpe, Maciej Liskiewicz, George T.H. Ellison. Robust causal inference using directed acyclic graphs: the R package 'dagitty'. International Journal of Epidemiology 45(6):1887-1894, 2016.

Supplementary Text 1: Specific funding sources and acknowledgements of participating studies

Study-specific funding sources

Genetics and Epidemiology of Colorectal Cancer Consortium (GECCO): National Cancer Institute, National Institutes of Health, U.S. Department of Health and Human Services (U01 CA164930, U01 CA137088, R01 CA059045, U01 CA164930, R01201407).
Genotyping/Sequencing services were provided by the Center for Inherited Disease Research (CIDR) (X01-HG008596 and X-01-HG007585). CIDR is fully funded through a federal contract from the National Institutes of Health to The Johns Hopkins University, contract number HHSN268201200008I. This research was funded in part through the NIH/NCI Cancer Center Support Grant P30 CA015704.

ASTERISK: a Hospital Clinical Research Program (PHRC-BRD09/C) from the University Hospital Center of Nantes (CHU de Nantes) and supported by the Regional Council of Pays de la Loire, the Groupement des Entreprises Françaises dans la Lutte contre le Cancer (GEFLUC), the Association Anne de Bretagne Génétique and the Ligue Régionale Contre le Cancer (LRCC).

The ATBC Study is supported by the Intramural Research Program of the U.S. National Cancer Institute, National Institutes of Health, and by U.S. Public Health Service contract HHSN261201500005C from the National Cancer Institute, Department of Health and Human Services.

COLO2&3: National Institutes of Health (R01 CA60987).
ColoCare: This work was supported by the National Institutes of Health (grant numbers R01 CA189184 (Li/Ulrich), U01 CA206110 (Ulrich/Li/Siegel/Figueireido/Colditz, 2P30CA015704-

40 (Gilliland), R01 CA207371 (Ulrich/Li)), the Matthias Lackas-Foundation, the German Consortium for Translational Cancer Research, and the EU TRANSCAN initiative.

The Colon Cancer Family Registry (CCFR) Illumina GWAS (“CCFR 1” and “CCFR 2”) was supported by funding from the National Cancer Institute, National Institutes of Health (grant numbers U01 CA122839, R01 CA143247 to G Casey). The Colon CFR/CORECT Affymetrix Axiom GWAS (“CCFR 3”) and OncoArray GWAS (“CCFR 4”) were supported by funding from National Cancer Institute, National Institutes of Health (grant number U19 CA148107 to S Gruber). The Colon CFR participant recruitment and collection of data and biospecimens used in this study were supported by the National Cancer Institute, National Institutes of Health (grant number UM1 CA167551) and through cooperative agreements with the following Colon CFR centers: Australasian Colorectal Cancer Family Registry (NCI/NIH grant numbers U01 CA074778 and U01/U24 CA097735), USC Consortium Colorectal Cancer Family Registry (NCI/NIH grant numbers U01/U24 CA074799), Mayo Clinic Cooperative Family Registry for Colon Cancer Studies (NCI/NIH grant number U01/U24 CA074800), Ontario Familial Colorectal Cancer Registry (NCI/NIH grant number U01/U24 CA074783), Seattle Colorectal Cancer Family Registry (NCI/NIH grant number U01/U24 CA074794), and University of Hawaii Colorectal Cancer Family Registry (NCI/NIH grant number U01/U24 CA074806), Additional support for case ascertainment was provided from the Surveillance.

Epidemiology and End Results (SEER) Program of the National Cancer Institute to Fred Hutchinson Cancer Research Center (Control Nos. N01-CN-67009 and N01-PC-35142, and Contract No. HHSN2612013000121), the Hawai‘i Department of Health (Control Nos. N01-PC- 67001 and N01-PC-35137, and Contract No. HHSN26120100037C, and the California Department of Public Health (contracts HHSN261201000035C awarded to the University of Southern California, and the following state cancer registries: AZ, CO, MN, NC, NH, and by the Victoria Cancer Registry and Ontario Cancer Registry.

COLON: The COLON study is sponsored by Wereld Kanker Onderzoek Fonds, including funds from grant 2014/1179 as part of the World Cancer Research Fund International Regular Grant Programme, by Alpe d'Huzes and the Dutch Cancer Society (UM 2012–5653, UW 2013-5927, UW2015-7946), and by TRANSCAN (JTC2012-MetaboCCC, JTC2013-FOCUS). The NQplus study is sponsored by a ZonMW investment grant (98-10030); by PREVIEW, the project PREVention of diabetes through lifestyle intervention and population studies in Europe and around the World (PREVIEW) project which received funding from the European Union Seventh Framework Programme (FP7/2007–2013) under grant no. 312057; by funds from TI Food and Nutrition (cardiovascular health theme), a public–private partnership on precompetitive research in food and nutrition; and by FOODBALL, the Food Biomarker Alliance, a project from JPI Healthy Diet for a Healthy Life.

Colorectal Transdisciplinary (CORECT) Study: The CORECT Study was supported by the National Cancer Institute, National Institutes of Health (NCI/NIH), U.S. Department of Health and Human Services (grant numbers U19 CA148107, R01 CA81488, P30 CA014089, R01 CA197350,; P01 CA196569; R01 CA201407) and National Institutes of Environmental Health Sciences, National Institutes of Health (grant number T32 ES013678).

CPS-II: The American Cancer Society funds the creation, maintenance, and updating of the Cancer Prevention Study-II (CPS-II) cohort. This study was conducted with Institutional Review Board approval.

DACHS: This work was supported by the German Research Council (BR 1704/6-1, BR 1704/6-3, BR 1704/6-4, CH 117/1-1, HO 5117/2-1, HE 5998/2-1, KL 2354/3-1, RO 2270/8-1 and BR 1704/17-1), the Interdisciplinary Research Program of the National Center for Tumor Diseases (NCT), Germany, and the German Federal Ministry of Education and Research (01KH0404, 01ER0814, 01ER0815, 01ER1505A and 01ER1505B).

DALS: National Institutes of Health (R01 CA48998 to M. L. Slattery).

EPIC funding: The coordination of EPIC is financially supported by the European Commission (DG-SANCO) and the International Agency for Research on Cancer. The national cohorts are supported by Danish Cancer Society (Denmark); German Cancer Aid, German Cancer Research Center (DKFZ), Federal Ministry of Education and Research (BMBF), Deutsche Krebshilfe, Deutsches Krebsforschungszentrum and Federal Ministry of Education and Research (Germany); the Hellenic Health Foundation (Greece); Associazione Italiana per la Ricerca sul Cancro-AIRC-Italy and National Research Council (Italy); Dutch Ministry of Public Health, Welfare and Sports (VWS), Netherlands Cancer Registry (NKR), LK Research Funds, Dutch Prevention Funds, Dutch ZON (Zorg Onderzoek Nederland), World Cancer Research Fund (WCRF), Statistics Netherlands (The Netherlands); Health Research Fund (FIS) PI13/00061 (EPIC-Granada) and, PI13/01162 (EPIC-Murcia), Regional Governments of Andalucía, Asturias, Basque Country, Murcia and Navarra, ISCIII Health Research Funds RD12/0036/0018 (cofounded by FEDER funds/European Regional Development Fund ERDF) (Spain); Swedish Cancer Society, Swedish Research Council and County Councils of Skåne and Västerbotten (Sweden); Cancer Research UK (14136 to EPIC-Norfolk; C8221/A19170 and C570/A16491 for EPIC-Oxford), Medical Research Council (1000143 to EPIC-Norfolk, MR/M012190/1 to EPIC-Oxford) (UK).

The KORA study was initiated and financed by the Helmholtz Zentrum München – German Research Center for Environmental Health, which is funded by the German Federal Ministry of Education and Research (BMBF) and by the State of Bavaria.

ESTHER/VERDI. This work was supported by grants from the Baden-Württemberg Ministry of Science, Research and Arts and the German Cancer Aid.

Harvard cohorts (HPFS, NHS, PHS): HPFS is supported by the National Institutes of Health

(P01 CA055075, UM1 CA167552, U01 CA167552, R01 CA137178, R01 CA151993, R35 CA197735, K07 CA190673, and P50 CA127003), NHS by the National Institutes of Health (R01 CA137178, P01 CA087969, UM1 CA186107, R01 CA151993, R35 CA197735, K07 CA190673, and P50 CA127003) and PHS by the National Institutes of Health (R01 CA042182).

Kentucky: This work was supported by the following grant support: Clinical Investigator Award from Damon Runyon Cancer Research Foundation (CI-8); NCI R01CA136726.

MCCS cohort recruitment was funded by VicHealth and Cancer Council Victoria. The MCCS was further supported by Australian NHMRC grants 509348, 209057, 251553 and 504711 and by infrastructure provided by Cancer Council Victoria. Cases and their vital status were ascertained through the Victorian Cancer Registry (VCR) and the Australian Institute of Health and Welfare (AIHW), including the National Death Index and the Australian Cancer Database.

MEC: National Institutes of Health (R37 CA54281, P01 CA033619, and R01 CA063464).

MECC: This work was supported by the National Institutes of Health, U.S. Department of Health and Human Services (R01 CA81488 to SBG and GR).

MSKCC: The work at Sloan Kettering in New York was supported by the Robert and Kate Niehaus Center for Inherited Cancer Genomics and the Romeo Milio Foundation. Moffitt: This work was supported by funding from the National Institutes of Health (grant numbers R01 CA189184, P30 CA076292), Florida Department of Health Bankhead-Coley Grant 09BN-13, and the University of South Florida Oehler Foundation. Moffitt contributions were supported in part by the Total Cancer Care Initiative, Collaborative Data Services Core, and Tissue Core at the H. Lee Moffitt Cancer Center & Research Institute, a National Cancer Institute-designated Comprehensive Cancer Center (grant number P30 CA076292).

NFCCR: This work was supported by an Interdisciplinary Health Research Team award from the Canadian Institutes of Health Research (CRT 43821); the National Institutes of Health, U.S. Department of Health and Human Serivces (U01 CA74783); and National Cancer Institute of Canada grants (18223 and 18226). The authors wish to acknowledge the contribution of Alexandre Belisle and the genotyping team of the McGill University and Génome Québec Innovation Centre, Montréal, Canada, for genotyping the Sequenom panel in the NFCCR samples. Funding was provided to Michael O. Woods by the Canadian Cancer Society Research Institute.

OFCCR: National Institutes of Health, through funding allocated to the Ontario Registry for Studies of Familial Colorectal Cancer (U01 CA074783); see CCFR section above. Additional funding toward genetic analyses of OFCCR includes the Ontario Research Fund, the Canadian Institutes of Health Research, and the Ontario Institute for Cancer Research, through generous support from the Ontario Ministry of Research and Innovation.

PLCO: Intramural Research Program of the Division of Cancer Epidemiology and Genetics and supported by contracts from the Division of Cancer Prevention, National Cancer Institute, NIH.

PMH: National Institutes of Health (R01 CA076366 to P.A. Newcomb).

SEARCH: The University of Cambridge has received salary support in respect of PDPP from the NHS in the East of England through the Clinical Academic Reserve. Cancer Research UK (C490/A16561); the UK National Institute for Health Research Biomedical Research Centres at the University of Cambridge.

SPAIN: The SPAIN study was supported by Instituto de Salud Carlos III, co-funded by FEDER funds –a way to build Europe– (grants PI14-613 and PI09-1286), Agency for Management of University and Research Grants (AGAUR) of the Catalan Government (grant 2017SGR723), and Junta de Castilla y León (grant LE22A10-2). Sample collection of this work was supported by the Xarxa de Bancs de Tumors de Catalunya sponsored by Pla Director d'Oncología de Catalunya (XBTC), Plataforma Biobancos PT13/0010/0013 and ICOBIOBANC, sponsored by the Catalan Institute of Oncology.

The Swedish Low-risk Colorectal Cancer Study: The study was supported by grants from the Swedish research council; K2015-55X-22674-01-4, K2008-55X-20157-03-3, K2006-72X-20157-01-2 and the Stockholm County Council (ALF project).

Swedish Mammography Cohort and Cohort of Swedish Men: This work is supported by the Swedish Research Council /Infrastructure grant, the Swedish Cancer Foundation, and the Karolinska Institute´s Distinguished Professor Award to Alicja Wolk.

UK Biobank: This research has been conducted using the UK Biobank Resource under Application Number 8614.

VITAL: National Institutes of Health (K05 CA154337).

WHI: The WHI program is funded by the National Heart, Lung, and Blood Institute, National Institutes of Health, U.S. Department of Health and Human Services through contracts HHSN268201100046C, HHSN268201100001C, HHSN268201100002C, HHSN268201100003C, HHSN268201100004C, and HHSN271201100004C.

CLUE II: This research was funded by the American Institute for Cancer Research and the Maryland Cigarette Restitution Fund at Johns Hopkins, and the NCI (P30 CA006973 to W.G. Nelson).

CORSA: “Österreichische Nationalbank Jubiläumsfondsprojekt” (12511) and Austrian Research Funding Agency (FFG) grant 829675.

Czech Republic CCS: This work was supported by the Grant Agency of the Czech Republic (grants CZ GA CR: GAP304/10/1286 and 1585) and by the Grant Agency of the Ministry of Health of the Czech Republic (grants AZV 15-27580A and AZV 17-30920A).

EDRN: This work is funded and supported by the NCI, EDRN Grant (U01 CA 84968-06).

EPICOLON: This work was supported by grants from Fondo de Investigación Sanitaria/FEDER (PI08/0024, PI08/1276, PS09/02368, P111/00219, PI11/00681, PI14/00173, PI14/00230, PI17/00509, 17/00878, Acción Transversal de Cáncer), Xunta de Galicia (PGIDIT07PXIB9101209PR), Ministerio de Economia y Competitividad (SAF07-64873, SAF 2010-19273, SAF2014-54453R), Fundación Científica de la Asociación Española contra el Cáncer (GCB13131592CAST), Beca Grupo de Trabajo “Oncología” AEG (Asociación Española de Gastroenterología), Fundación Privada Olga Torres, FP7 CHIBCHA Consortium, Agència de Gestió d’Ajuts Universitaris i de Recerca (AGAUR, Generalitat de Catalunya, 2014SGR135, 2014SGR255, 2017SGR21, 2017SGR653), Catalan Tumour Bank Network (Pla Director d’Oncologia, Generalitat de Catalunya), PERIS (SLT002/16/00398, Generalitat de Catalunya), CERCA Programme (Generalitat de Catalunya) and COST Action BM1206. CIBERehd is funded by the Instituto de Salud Carlos III.

Hawaii Adenoma Study: NCI grants R01 CA72520.

HCES-CRC: the Hwasun Cancer Epidemiology Study–Colon and Rectum Cancer (HCES-CRC; grants from Chonnam National University Hwasun Hospital, HCRI15011-1).

LCCS: The Leeds Colorectal Cancer Study was funded by the Food Standards Agency and Cancer Research UK Programme Award (C588/A19167).

NCCCS I & II: We acknowledge funding support for this project from the National Institutes of Health, R01 CA66635 and P30 DK034987.

NSHDS: Swedish Research Council; Swedish Cancer Society; Cutting-Edge Research Grant and other grants from the County Council of Västerbotten, Sweden; Wallenberg Centre for Molecular Medicine at Umeå University; Lion's Cancer Research Foundation at Umeå University; the Cancer Research Foundation in Northern Sweden; and the Faculty of Medicine, Umeå University, Umeå, Sweden.

OSUMC: OCCPI funding was provided by Pelotonia and HNPCC funding was provided by the NCI (CA16058 and CA67941).

SELECT: The Selenium and Vitamin E Cancer Prevention Trial (SELECT) was supported by the National Cancer Institute of the National Institutes of Health under Award Numbers UM1CA182883 and U10CA37429.

SMS: This work was supported by the National Cancer Institute (grant P01 CA074184 to J.D.P. and P.A.N., grants R01 CA097325, R03 CA153323, and K05 CA152715 to P.A.N., and the National Center for Advancing Translational Sciences at the National Institutes of Health (grant KL2 TR000421 to A.N.B.-H.)

Acknowledgements

We thank Sandra Reitinger and Lina Maqboul for supporting UCB measurement in lab.

ASTERISK: We are very grateful to Dr. Bruno Buecher without whom this project would not have existed. We also thank all those who agreed to participate in this study, including the patients and the healthy control persons, as well as all the physicians, technicians and students.

COLON and NQplus: the authors would like to thank the COLON and NQplus investigators at Wageningen University & Research and the involved clinicians in the participating hospitals.

CPS-II: The authors thank the CPS-II participants and Study Management Group for their invaluable contributions to this research. The authors would also like to acknowledge the contribution to this study from central cancer registries supported through the Centers for Disease Control and Prevention National Program of Cancer Registries, and cancer registries supported by the National Cancer Institute Surveillance Epidemiology and End Results program.

DACHS: We thank all participants and cooperating clinicians, and Ute Handte-Daub, Utz Benscheid, Muhabbet Celik and Ursula Eilber for excellent technical assistance.

Harvard cohorts: The study protocol was approved by the institutional review boards of the Brigham and Women's Hospital and Harvard T.H. Chan School of Public Health, and those of participating registries as required. We would like to thank the participants and staff of the HPFS, NHS, and PHS for their valuable contributions as well as the following state cancer registries for their help: AL, AZ, AR, CA, CO, CT, DE, FL, GA, ID, IL, IN, IA, KY, LA, ME, MD, MA, MI, NE, NH, NJ, NY, NC, ND, OH, OK, OR, PA, RI, SC, TN, TX, VA, WA, WY. The authors assume full responsibility for analyses and interpretation of these data.

HPFS, NHS and PHS for their valuable contributions as well as the following state cancer registries for their help: AL, AZ, AR, CA, CO, CT, DE, FL, GA, ID, IL, IN, IA, KY, LA, ME, MD, MA, MI, NE, NH, NJ, NY, NC, ND, OH, OK, OR, PA, RI, SC, TN, TX, VA, WA, WY.
The authors assume full responsibility for analyses and interpretation of these data. Kentucky: We would like to acknowledge the staff at the Kentucky Cancer Registry.

PLCO: The authors thank the PLCO Cancer Screening Trial screening center investigators and the staff from Information Management Services Inc and Westat Inc. Most importantly, we thank the study participants for their contributions that made this study possible.

PMH: The authors would like to thank the study participants and staff of the Hormones and Colon Cancer study.

SEARCH: We thank the SEARCH team.

WHI: The authors thank the WHI investigators and staff for their dedication, and the study participants for making the program possible. A full listing of WHI investigators can be found at:
http://www.whi.org/researchers/Documents%20%20Write%20a%20Paper/WHI%20Investigator%20Short%20List.pdf

We acknowledge COMPASS (Comprehensive Center for the Advancement of Scientific Strategies) and Deanna Stelling, Mark Thornquist, and Greg Warnick at the Fred Hutchinson Cancer Research Center for their work harmonizing the GECCO epidemiological data set.

CLUE II: We thank Judith Hoffman-Bolton, Senior Research Program Coordinator, for her contributions to the conduct of CLUE and its participation in this study. We also thank the participants in CLUE.

CORSA: We kindly thank all those who contributed to the screening project Burgenland against CRC. Furthermore, we are grateful to Doris Mejri and Monika Hunjadi for laboratory assistance.

Czech Republic CCS: We are thankful to all clinicians in major hospitals in the Czech Republic, without whom the study would not be practicable. We are also sincerely grateful to all patients participating in this study.

EDRN: We acknowledge all the following contributors to the development of the resource: University of Pittsburgh School of Medicine, Department of Gastroenterology, Hepatology and Nutrition: Lynda Dzubinski; University of Pittsburgh School of Medicine, Department of Pathology: Michelle Bisceglia; and University of Pittsburgh School of Medicine, Department of Biomedical Informatics.

EPICOLON: We are sincerely grateful to all patients participating in this study who were recruited as part of the EPICOLON project. We acknowledge the Spanish National DNA Bank, Biobank of Hospital Clínic–IDIBAPS and Biobanco Vasco for the availability of the samples. The work was carried out (in part) at the Esther Koplowitz Centre, Barcelona.

LCCS: We acknowledge the contributions of Jennifer Barrett, Robin Waxman, Gillian Smith and Emma Northwood in conducting this study.

NCCCS I & II: We would like to thank the study participants, and the NC Colorectal Cancer Study staff.

NSHDS: We thank all participants in the NSHDS cohorts, the staff at the Department of Biobank Research, Umeå University, the staff at Biobanken norr, Västerbotten County

Council, the scientists managing the Northern Sweden Diet Database.

SELECT: We thank the research and clinical staff at the sites that participated on SELECT study, without whom the trial would not have been successful. We are also grateful to the 35 533 dedicated men who participated in SELECT.
